# PDGFB targeting biodegradable FePt alloy assembly for MRI guided starvation-enhancing chemodynamic therapy of cancer

**DOI:** 10.1186/s12951-022-01482-x

**Published:** 2022-06-07

**Authors:** Caiyun Zhang, Zhiguo Leng, Yinfeng Wang, Lang Ran, Xia Qin, Huan Xin, Xiaotong Xu, Guilong Zhang, Zhaowei Xu

**Affiliations:** School of Pharmacy, Shandong Technology Innovation Center of Molecular Targeting and Intelligent Diagnosis and Treatment, Binzhou Medical University, Yantai, 264003 People’s Republic of China

**Keywords:** PDGFB, Magnetic resonance imaging, GOx, starvation therapy, Chemodynamic therapy, Chemotherapy

## Abstract

The application of chemodynamic therapy (CDT) for cancer is a serious challenge owing to the low efficiency of the Fenton catalyst and insufficient H_2_O_2_ expression in cells. Herein, we fabricated a PDGFB targeting, biodegradable FePt alloy assembly for magnetic resonance imaging (MRI)-guided chemotherapy and starving-enhanced chemodynamic therapy for cancer using PDGFB targeting, pH-sensitive liposome-coated FePt alloys, and GOx (pLFePt-GOx). We found that the Fenton-catalytic activity of FePt alloys was far stronger than that of traditional ultrasmall iron oxide nanoparticle (UION). Upon entry into cancer cells, pLFePt-GOx nanoliposomes degraded into many tiny FePt alloys and released GOx owing to the weakly acidic nature of the tumor microenvironment (TME). The released GOx-mediated glucose consumption not only caused a starvation status but also increased the level of cellular H_2_O_2_ and acidity, promoting Fenton reaction by FePt alloys and resulting in an increase in reactive oxygen species (ROS) accumulation in cells, which ultimately realized starving-enhanced chemodynamic process for killing tumor cells. The anticancer mechanism of pLFePt-GOx involved ROS-mediated apoptosis and ferroptosis, and glucose depletion-mediated starvation death. In the in vivo assay, the systemic delivery of pLFePt-GOx showed excellent antitumor activity with low biological toxicity and significantly enhanced T_2_-weighted magnetic resonance imaging (MRI) signal of the tumor, indicating that pLFePt-GOx can serve as a highly efficient theranostic tool for cancer. This work thus describes an effective, novel multi-modal cancer theranostic system.

**Supplementary Information:**

The online version contains supplementary material available at 10.1186/s12951-022-01482-x.

## Introduction

Cancer is a threatening challenge to human health worldwide [1–3]. The rapid proliferation and differential metabolism occurring in cancer tissues result in the formation of a hypoxic and weakly acidic tumor microenvironment (TME) [4, 5]. These characteristics are closely associated with cancer metastasis and multidrug resistance, which restricts the therapeutic efficiency of conventional chemotherapeutic drugs [6, 7]. Furthermore, the accuracy of diagnosing malignancy in the early stage is poor, resulting in the disease being highly lethal and having a poor prognosis. However, with the development of nanotechnology, it is now possible to integrate a therapeutic drug and advanced diagnostic agent into a single system for early diagnosis and effective therapy of cancer [8–10]. Furthermore, the early diagnosis and efficiency of cancer treatment can be promoted by developing a TME-responsive theranostic system.

Recently, heavy metal ions including iron [11], copper [12], and manganese [13], have been utilized to catalyze the conversion of endogenous hydrogen peroxide (H_2_O_2_) to highly toxic hydroxyl radicals (·OH), whose accumulation then triggers reactive oxygen species (ROS)-mediated oxidative stress and non-programmed cell apoptosis; this process is termed chemodynamic therapy (CDT) [14–17]. Recently, Tao’s group reported that surface-oxidized arsenene nanosheets with oxygen vacancies also catalyzed a Fenton-like reaction, and then generate ·OH and O_2_ from H_2_O_2_ [18], which provided a completely new concept for developing highly efficient CDT agent. Ultrasmall iron oxide nanoparticles (UION) have widely been utilized in CDT and diagnostic imaging of cancer owing to their high catalytic activity and ability to enhance T_2_ magnetic resonance imaging (MRI) contrast [19, 20]. However, UION-based CDT is still incapable of inducing cancer cell death. This is mainly due to the following two reasons: (1) Fenton catalytic efficacy of UION is only effective under highly acidic conditions [21]. In weakly acidic TME, the CDT induced by the UION-mediated Fenton reaction is relatively inefficient and insufficient to effectively inhibit tumor growth [22, 23]. (2) CDT mainly relies on Fenton's activity to convert intracellular H_2_O_2_ into highly toxic hydroxyl radicals and thus damage the tumor cell [24]. Although cancer cells express excessive H_2_O_2_ compared to normal cells, the H_2_O_2_ level in cancer cells is still too low to effectively activate CDT for inducing cancer cell death [25]. Therefore, the development of a novel strategy based on Fe-based nanomaterials to increase endogenous H_2_O_2_ and enhance the catalytic activity of CDT agents will help improve clinical therapy and diagnosis of cancer.

Considering the rapid proliferative phenotype and increased glucose consumption of cancer cells, cutting off the nutrient supply to induce starvation status is a promising strategy for tumor suppression [26–28]. Glucose oxidase (GOx), widely used in the food industry, can decompose glucose into gluconic acid and H_2_O_2_ in the presence of oxygen [29, 30]. When successfully delivered to the tumor cells, GOx triggers simultaneous glucose and oxygen deprivation, which dramatically increases cellular acidity and the H_2_O_2_ level. This process not only realizes starvation therapy of tumor but also promotes the Fenton catalytic efficiency, thereby strengthening tumor CDT owing to the sufficient supplementation of H_2_O_2_. However, non-targeting GOx-mediated starvation therapy may result in the distribution of GOx into normal tissue, which may further lead to oxidization of glucose and cause potential systematic toxicity to the body [31, 32]; this severely limits its clinical application. Besides, systemic distribution of excessive ROS induce more serious DNA damage for normal cells, resulting in oxidative toxicity for normal organs, especially for the intestinal tissue [33, 34]. Thus, it is urgent to develop a tumor-targeting GOx and Fenton catalyst delivery system to achieve highly efficient CDT and safe starvation therapy for cancer.

PDGFB/PDGFR-β axis is well documented as a vital oncogenic signal and potential therapeutic target for different types of carcinomas, such as prostate cancer [35], pancreatic cancer [36], and breast cancer [37]. Overexpression of PDGFB has been detected in several human malignancies including pancreatic cancer, gastric cancer, glioma, and melanoma [38–40]. Moreover, the expression variability of PDGFB and its cognate receptor were found to be closely associated with lymph node metastasis and outcomes in patients [41]. For example, gastric cancer patients with lymph node metastasis had high levels of PDGFB and PDGFRβ [42]. In addition, PDGFB/PDGFRβ were found to be highly expressed in many subtypes of breast cancer cells and tissues, and breast cancer-associated stromal cells and vasculature also exhibited positive staining of PDGFB/PDGFRβ [43]. These studies indicated that PDGFB/PDGFRβ axis plays an important role in tumorigenesis and cancer progression; thus it can serve as a promising prognostic parameter and therapeutic drug target.

**Scheme 1.**
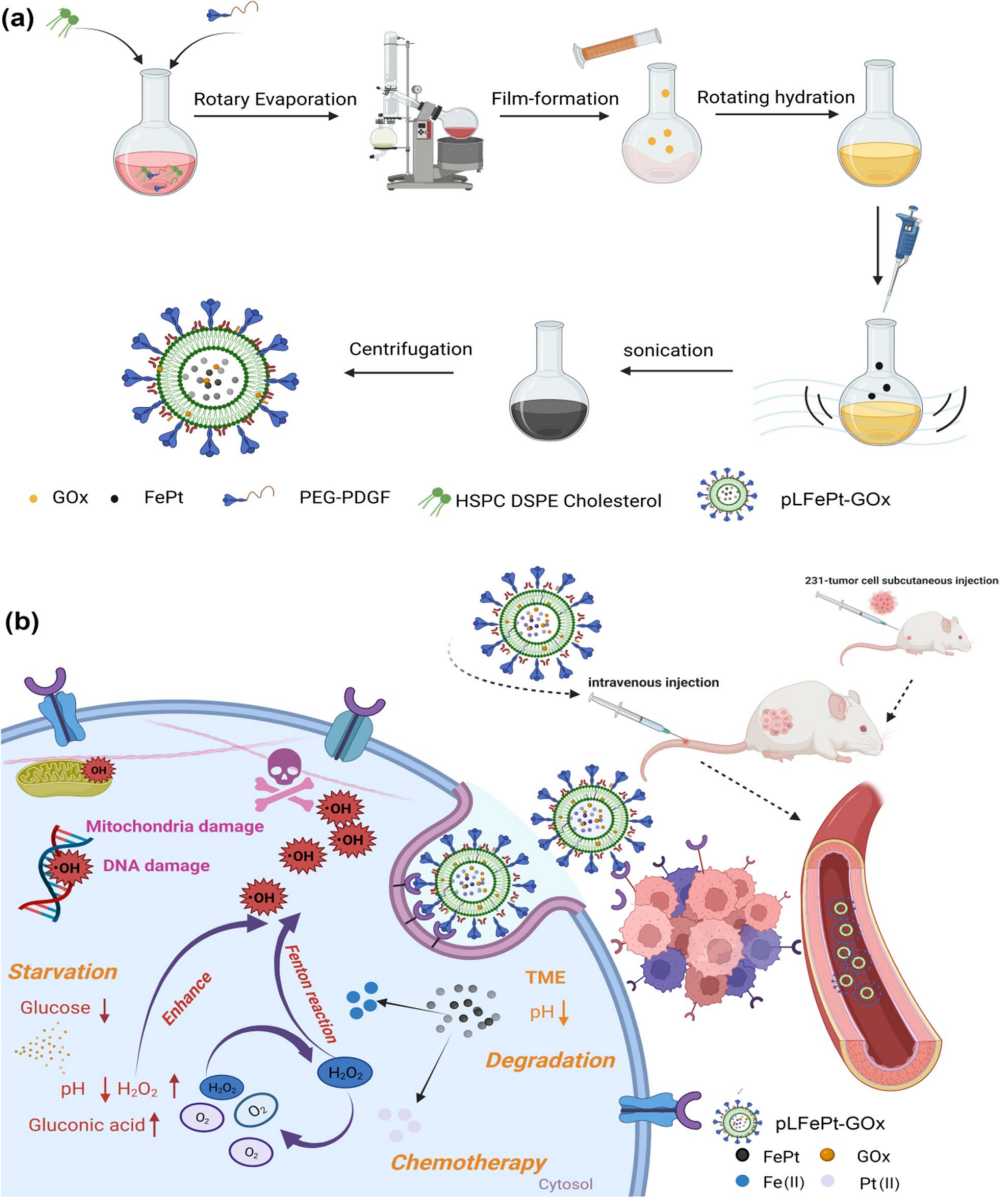
Schematic illustration of **a** the preparation of pLFePt-GOx and **b** the mechanism by which it increases the efficacy of starvation-enhancing cancer CDT

In this study, we found that FePt alloys possessed stronger Fenton-catalytic activity than UION, and could be an ideal Fenton catalyst. Based on this, we fabricated a PDGFB targeting and biodegradable FePt alloy assembly for MRI-guided chemotherapy and starving-enhanced chemodynamic therapy of cancer using PDGFB targeting, pH-sensitive liposome-coated FePt alloys, and GOx (pLFePt-GOx) (Scheme 1). Upon entry into cancer cells, pLFePt-GOx nanoliposomes degraded into many tiny FePt alloys owing to the weakly acidic nature of TME (Scheme 1b). Subsequently, these ultrasmall FePt alloys not only catalyzed endogenous H_2_O_2_ into highly toxic ·OH but also released abundant Pt(II) triggered by ROS, contributing to chemotherapy and CDT. In addition, the released GOx consumed cellular glucose and produced gluconic acid and H_2_O_2_, contributing to starvation therapy and accelerating CDT. Besides, the pLFePt-GOx nanoliposome possessed excellent superparamagnetism, and its targeted delivery significantly enhanced the T_2_-weighted MRI signal of the tumor, which is beneficial for achieving high-quality MR images and realizing accurate diagnosis of tumor. Therefore, this work describes an effective, novel multi-modal theranostic system for cancer.

## Results and discussion

### Morphology observation of pLFePt-GOx

**Fig. 1:**
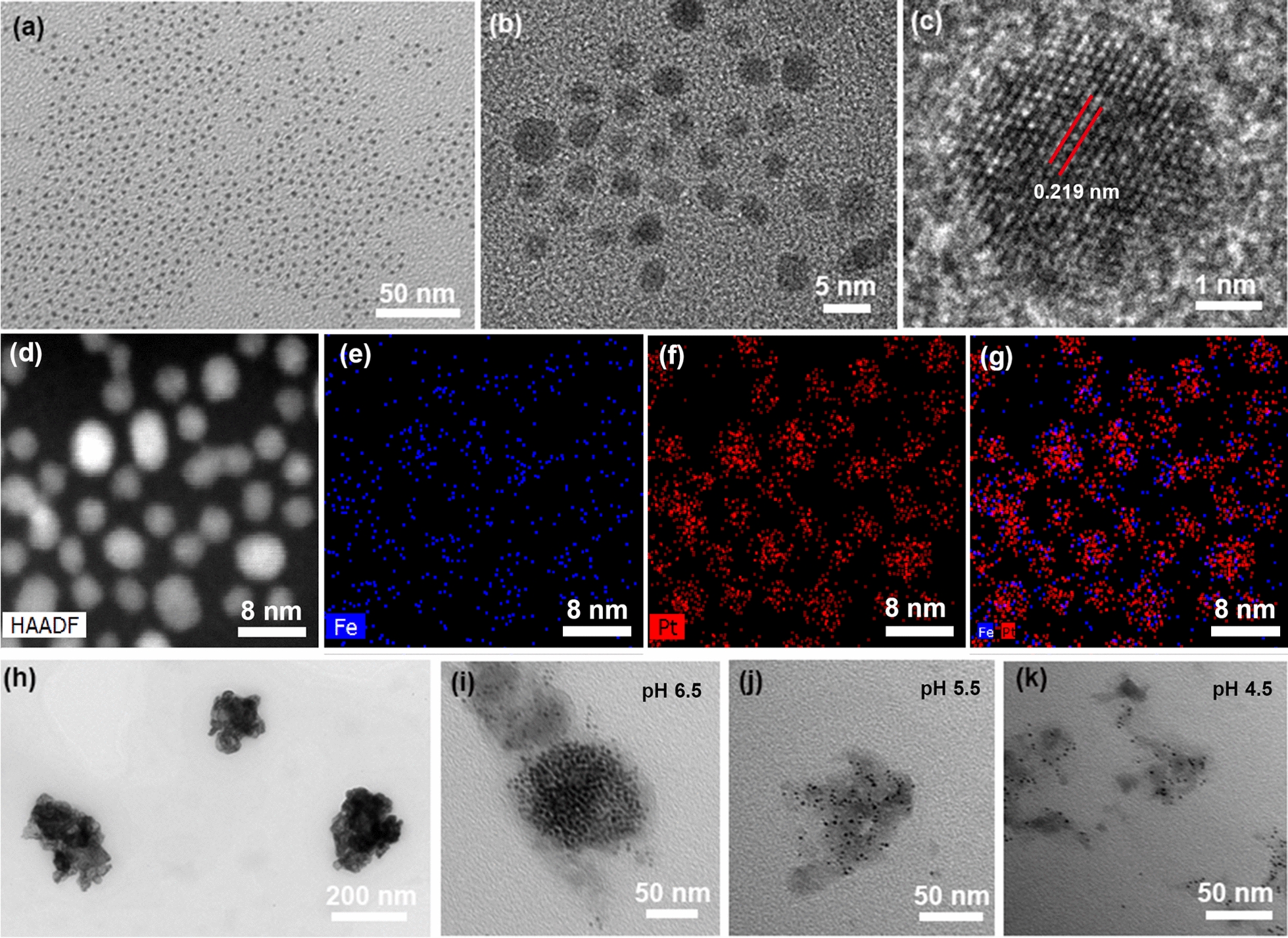
TEM observation of FePt nanoparticles: **a** low-magnification image, **b** high-magnification image, **c** high-resolution image. Elemental mapping images of FePt nanoparticles: **d** bright field image, **e** Fe element, **f** Pt element, and **g** merged images. **h** TEM images of LFePt-GOx. **i**–**k** TEM images of LFePt-GOx after treatment with different acidic solutions (pH 6.5, 5.5, and 4.5), respectively

The ultra-small FePt alloys were synthesized using ferric acetylacetonate and platinum bis(acetylacetonate) as precursors via a modified high-temperature decomposition method. The prepared FePt alloys were hydrophobic and only dispersed in n-hexane solution. For their in vivo application, hydrophobic FePt alloys were converted to hydrophilic FePt alloys through ligand exchange. Additional file 1: Fig. S1 shows that the synthesized hydrophilic FePt alloys had excellent colloidal stability in an aqueous solution. Furthermore, these hydrophilic FePt alloys were observed via transmission electronic microscopy (TEM). As shown in Fig. 1a, the FePt alloys were very small and showed excellent monodispersibility. Subsequently, the magnified images indicated that the size of FePt alloys mainly ranged from 2–4 nm (Fig. 1b). The high-resolution TEM images showed significant lattice fringe, and interplanar spacing was 0.219, consistent with the (111) plane of FePt alloys (Fig. 1c). The elemental mapping analysis confirmed that Fe and Pt elements were uniformly distributed in alloys (Fig. 1d–g). Together, these results demonstrated that ultrasmall FePt alloys were successfully synthesized. Subsequently, these FePt alloys and GOx were simultaneously coated by PDGFB targeting, pH-sensitive liposome to fabricate pLFePt-GOx nanoliposomes. As shown in Fig. 1h and Additional file 1: Fig. S2, pLFePt-GOx nanoliposomes showed significant assembly of ultrasmall FePt alloys and were 200 nm in size. In addition, significant liposomal layer was observed around the surface of assembly, confirming the successful fabrication of pLFePt-GOx. To verify their pH sensitiveness, pLFePt-GOx nanoliposomes were treated with different pH solutions. With the decrease in pH, pLFePt-GOx nanoliposomes gradually split into many tiny crystals, indicating that pLFePt-GOx had excellent responsive ability to weakly acidic TME (Fig. 1h–k).

### Physico-chemical property of pLFePt-GOx

**Fig. 2:**
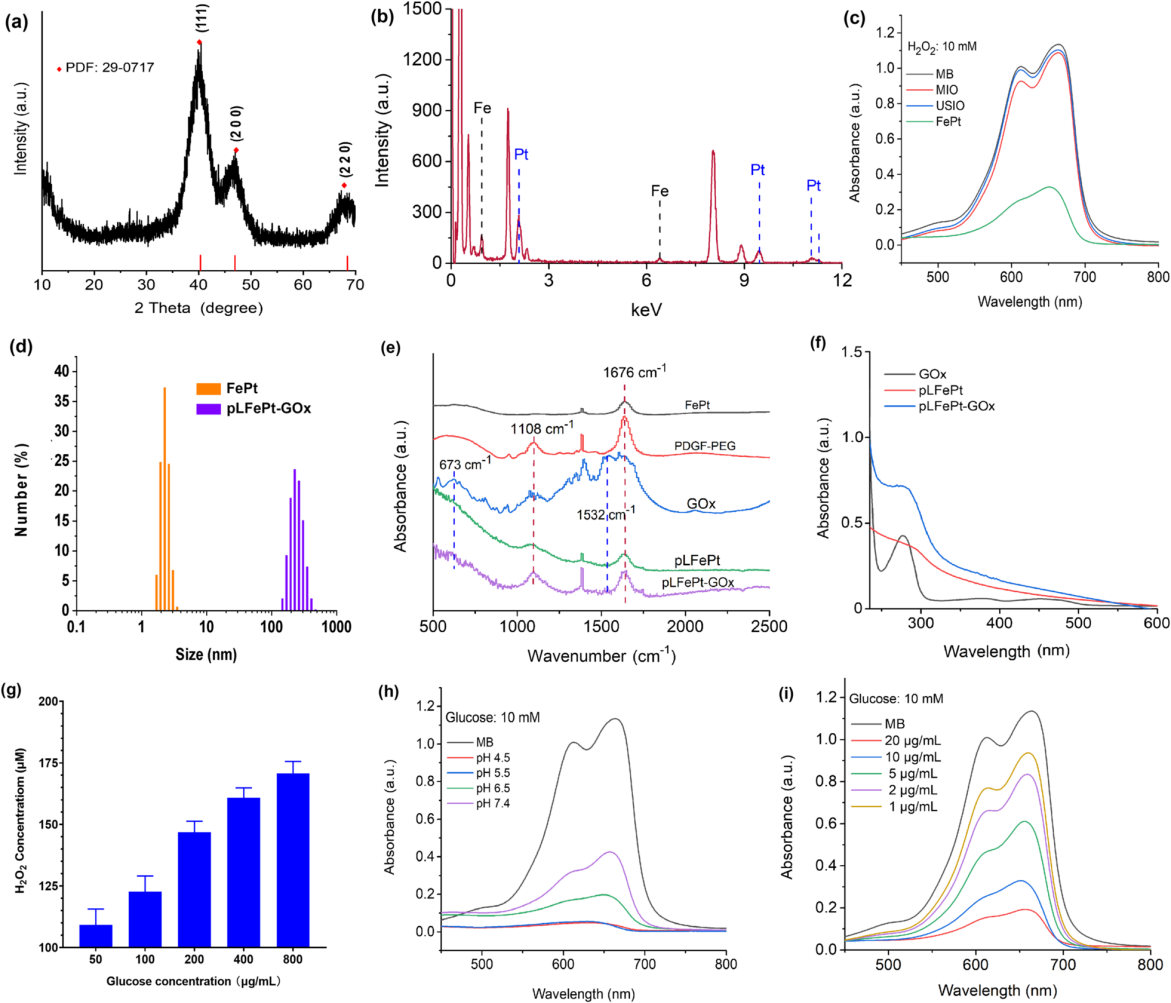
**a** XRD spectra of FePt nanoparticles. **b** EDX spectra of FePt nanoparticles. **c** The degradation curves of MB solution containing 10 mM of H_2_O_2_ after MIO, USIO, and FePt treatment. **d** Hydrodynamic particle size of FePt and pLFePt-GOx. **e** FT-IR spectra of FePt, GOx, PDGFB-PEG, pLFePt, and pLFePt-GOx. **f** Uv–vis spectra of pLFePt before and after loading GOx. **g** H_2_O_2_ production in different concentrations of glucose solution catalyzed by GOx. (h) MB degradation curves of pLFePt-GOx in different pH solutions containing 10 mM of glucose. **i** MB degradation curves of different concentrations of pLFePt-GOx in 10 mM of glucose solution at pH 4.5

The crystal structure of FePt alloy was characterized via x-ray diffraction (XRD) spectra. As shown in Fig. 2a, the spectra showed significant diffraction peaks at 41°, 48°, and 68°, which were assigned to the crystal structure of FePt alloys (PDF:#29–0717). Furthermore, energy dispersive X-ray (EDX) spectra confirmed the presence of Fe and Pt species (Fig. 2b). Together, these results confirmed the successful synthesis of FePt alloys. To confirm the presence of Fenton reaction, electron spin resonance (ESR) spectra was employed as direct evidence to validate the generation of ·OH, by applying 5,5-dimethyl-1-pyrroline-N-oxide (DMPO) as a ·OH capture probe. After FePt alloy treatment, the ESR spectra of H_2_O_2_ solution showed significant 1:2:2:1 characteristic peaks assigned to ·OH (Additional file 1: Fig. S3), confirming the ability of FePt alloy to produce ·OH. In addition, compared to traditional Fenton catalyst (MIO or USIO), the H_2_O_2_ solution treated with FePt alloys showed the strongest ·OH signals, indicating that the FePt alloys as CDT agent possessed the best Fenton-catalytic activity. In addition, methylene blue (MB) can be also degraded by ·OH, resulting in the formation of a colorless solution. Based on this phenomenon, the MB degradation experiment was further used to assess the catalytic activity of FePt alloys. As shown in Fig. 2c, the mixed solution of MB and H_2_O_2_ treated with magnetic iron oxide (MIO) and ultrasmall iron oxide (USIO) only showed a slight decrease in absorbance; In comparison to this mixed solution, the MB solution containing FePt alloys exhibited a very low absorbance, implying that FePt alloys could effectively catalyze H_2_O_2_ to ·OH, thus promoting MB degradation. In addition, the degraded ratio of MB was quantitatively analyzed using a linear fit standard curve (Additional file 1: Fig. S4a). The result indicated that the degraded ratios of MB solution treated with USIO, MIO, and FePt alloys were 3.67%, 5.71%, and 98.39%, respectively (Additional file 1: Fig. S4b). This result demonstrated that the Fenton-catalytic activity of FePt alloys is far higher than that of the traditional CDT agent. Therefore, FePt alloy might serve as an ideal and highly efficient CDT agent for tumor therapy.

Although FePt alloys possess ultrahigh catalytic activity, their ultrasmall size may cause their excretion from the body, which severely limits their application in vivo. Besides, the efficacy of tumor CDT not only depends on the activity of the catalyst but is also closely associated with cellular H_2_O_2_ level and acidity. To overcome these limitations, glucose oxidase (GOx), which catalyzes glucose into gluconic acid and H_2_O_2_ in the presence of oxygen, was integrated with FePt alloys via PDGFB targeting liposomes to form the pLFePt-GOx assembly. As shown in Fig. 2d, the average hydrodynamic size of ultrasmall FePt alloys was only approximately 2.5 nm; however, pLFePt-GOx had a size of approximately 240 nm. Furthermore, it was noted that pLFePt-GOx maintained the narrow hydrodynamic size distribution, indicating that pLFePt-GOx possessed excellent colloidal performance. Subsequently, the colloidal stability of pLFePt-GOx was investigated by observing hydrodynamic size change under different media containing phosphate buffer saline (PBS) and fetal bovine serum (FBS) (Additional file 1: Fig. S5). It could be seen that the hydrodynamic size of pLFePt-GOx had no significant variation during standing for 48 h, and the corresponding particle dispersity index (PDI) kept at the range of 0.3–0.6, indicating that pLFePt-GOx solution had excellent colloidal stability. Subsequently, fourier transform infrared (FT-IR) spectra were used to analyze the fabrication process of pLFePt-GOx (Fig. 2e). Compared to the spectra of FePt alloys, the spectra of pLFePt-GOx showed some new peaks at 673, 1532, 1108, and 1676 cm^−1^, which were assigned to C-N bending vibration and C-N stretching vibration of GOx and C-O stretching vibration and C = O stretching vibration of PDGFB-PEG, respectively. This result confirmed that GOx and FePt alloys had been successfully assembled into pLFePt-GOx through PDGFB targeting liposomes. X-ray photoelectron spectroscopy (XPS) spectra analysis was used to further analyze the composition of pLFePt-GOx (Additional file 1: Fig. S6). The spectra confirmed the presence of Fe, Pt, P, and C elements (Additional file 1: Fig. S6a). The peaks of Pt4f appeared at 74.1 eV and 70.4 eV, indicating that platinum in FePt alloy was almost in a zero valent state (Additional file 1: Fig. S6c). In addition, Fe2p peaks appeared at 706.7 eV, 710.1 eV, and 713.3 eV which were assigned to Fe(0), Fe(II), and Fe(III), respectively (Additional file 1: Fig. S6b). This result indicated that the zero valence Fe detected in FePt alloys had been partly oxidized after loading the alloy into the assembly. In addition, the appearance of the P2p peak, which originated from liposomes, further confirmed the successful fabrication of pLFePt-GOx (Additional file 1: Fig. S6d). The GOx loading into pLFePt-GOx was also monitored through Uv–vis spectra. As shown in Fig. 2f, pure GOx showed significant absorption at a wavelength of 256 nm, whereas pLFePt had no absorption at the same wavelength. Notably, after loading GOx, the broad peak of pLFePt-GOx appeared at the wavelength of 265 nm, further confirming the successful loading of GOx. Besides, the loading content of GOx and FePt alloys in pLFePt-GOx were analyzed using Uv–Vis spectra and ICP-MS analysis, respectively. The result indicated that the loading content of GOx and FePt alloys in pLFePt-GOx was 24.6% and 41.08%, respectively.

To observe the ability of pLFePt-GOx to catalyze glucose into gluconic acid and H_2_O_2_, we explored H_2_O_2_ levels and acidity in the glucose solution after pLFePt-GOx treatment. As shown in Fig. 2g and Additional file 1: Fig. S7, the H_2_O_2_ content and acidity of the solution gradually increased with the increase of glucose concentration, indicating that the ability of pLFePt-GOx to produce H_2_O_2_ is dependent on glucose concentration. This result demonstrated that pLFePt-GOx could be an excellent agent to achieve elevated cellular H_2_O_2_ levels and decreased cellular acidity, which dramatically promotes cancer CDT. In addition, pLFePt-GOx could effectively degrade MB in presence of glucose, and showed pH- and concentration-dependent catalytic activity (Fig. 2h, i), indicating that the integration of FePt alloys and GOx can synergistically enhance cancer CDT. Pt(II) coordinates with DNA during chemotherapy. To verify the anticancer potential of FePt alloys, the release of Pt(II) from pLFePt-GOx was investigated via inductively coupled plasma-mass spectrometry (ICP-MS) analysis. The results showed that pLFePt-GOx released a certain amount of Pt(II) under weakly acidic conditions (pH 4.5) (Additional file 1: Fig. S8). Moreover, with H_2_O_2_ treatment, the Pt(II) released from pLFePt-GOx significantly accelerated, indicating that Pt(II) release was closely associated with ROS production. These results demonstrate that pLFePt-GOx possesses a promising potential for inducing cancer cell death via chemotherapy.

### Cell internalization, cellular ROS activation and H_2_O_2_ production

**Fig. 3:**
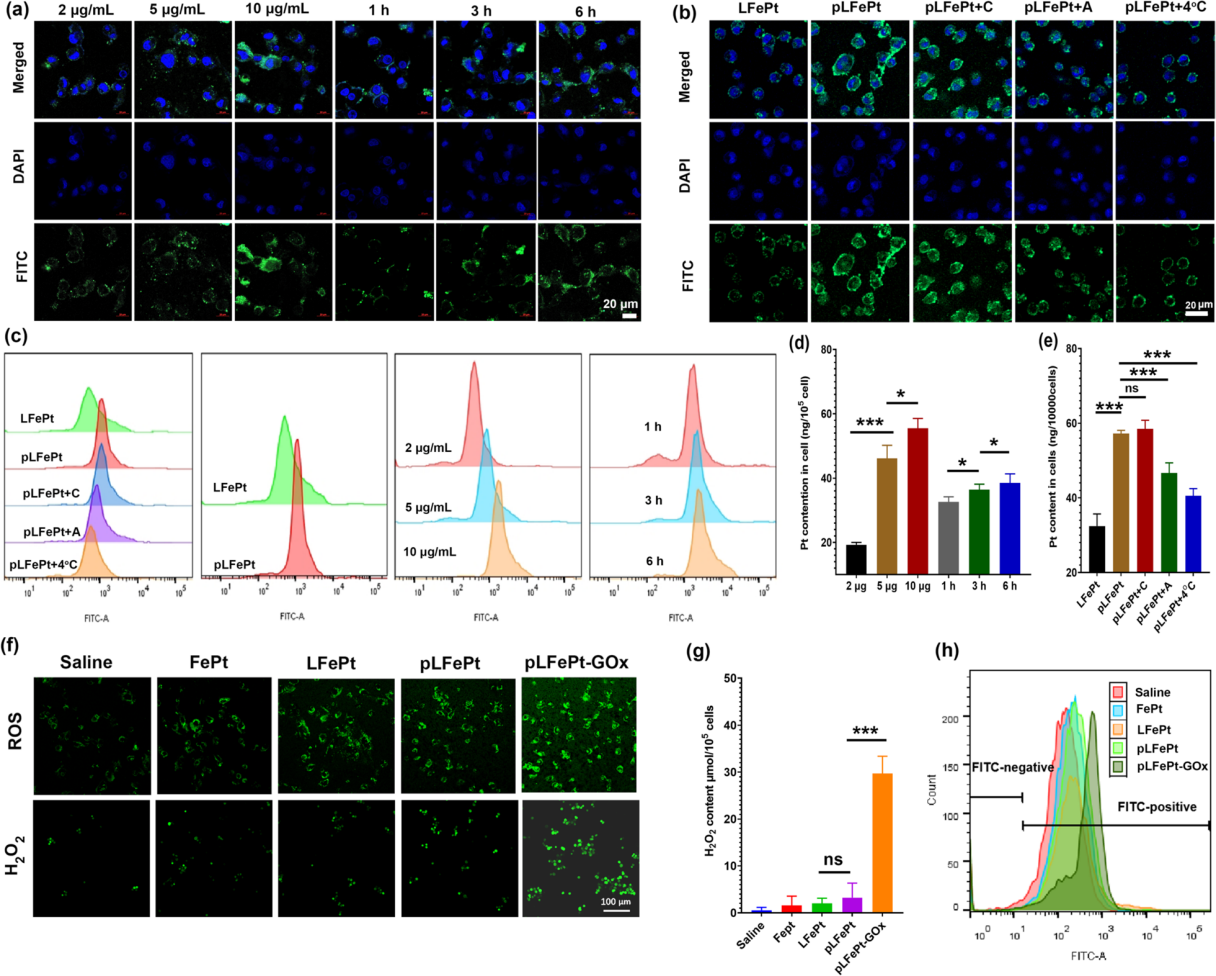
The internalization process of MDA-MB-231 cells: **a** CLSM observation after different concentrations of pLFePt treatment for different time; **b** CLSM observation after different samples treatment. **c** Corresponding flow cytometry analysis and **d**, **e** ICP-MS analysis for the internalization process. ROS and H_2_O_2_ production of MDA-MB-231 cells treated with different samples: **f** CLSM observation, **g** H_2_O_2_ detection kit, **h** flow cytometry analysis for cellular ROS

To evaluate the tumor-targeting effect of pLFePt and reveal how the nanotheranostic agent enters the cells, the internalization process of FITC-labeled pLFePt was observed by CLSM. As shown in Fig. 3a, the intensity of green fluorescence of MDA-MB-231 cells gradually increased with an increase in incubation time and concentration of pLFePt. Moreover, the green fluorescence of MDA-MB-231 cells treated with pLFePt was significantly stronger than that of cells treated with LFePt, indicating the excellent targeting ability of pLFePt dramatically enhanced its uptake by cells (Fig. 3b). Furthermore, to illustrate the internalization mechanism of pLFePt, MDA-MB-231 cells were incubated with the endocytic inhibitors and pLFePt. As shown in Fig. 3b, macropinocytosis inhibitor (amiloride) and low temperature significantly decreased the cellular uptake of pLFePt, while the inhibitor of clathrin-mediated endocytosis (chlorpromazine) had negligibly decreased this uptake. Meanwhile, flow cytometry analysis of Fig. 3c showed the similar results with CLSM observation. Subsequently, the cellular uptake of pLFePt was quantitatively analyzed using ICP-MS. The results demonstrated that Pt content in cells treated with pLFePt gradually increased with the increase in incubation dose and time, showing time- and dosage-dependent tendency (Fig. 3d). Remarkably, Pt content in pLFePt-treated cells reached up to 56.4 ng/10000cells, which was more two-fold than that in LFePt-treated cells. This result further illustrated excellent targeting of pLFePt to MDA-MB-231 cells. Furthermore, co-incubation with chlorpromazine did not decrease Pt content in cells; however, amiloride and low temperature significantly decreased cellular Pt content (Fig. 3e). According to the mentioned analysis, it could be concluded that the internalization process of pLFePt is an energy-consuming pinocytosis effect.

Upon entry into cells, pLFePt-GOx splits into many tiny FePt alloys and releases GOx owing to the weakly acidic TME. The released GOx depletes glucose and produces H_2_O_2_, thus enhancing the H_2_O_2_ level and acidity in cells. Considering the above-mentioned phenomenon, H_2_O_2_ levels in MDA-MB-231 cells treated with pLFePt-GOx were investigated using an H_2_O_2_ detection kit and fluorescence probe. As shown in Fig. 3f, g, after FePt, LFePt, and pLFePt treatment, H_2_O_2_ levels in treated MDA-MB-231 cells did not significantly vary among the treatment groups, indicating that MDA-MB-231 cells had a certain ability to maintain the balance of redox-reduction substance. Notably, the H_2_O_2_ level in MDA-MB-231 cells treated with pLFePt-GOx sharply increased compared to other groups, suggesting the misbalance of redox in these cells. This result demonstrated that pLFePt-GOx indeed significantly elevates cellular H_2_O_2_ level, and this elevated H_2_O_2_ can further trigger ROS production via FePt alloy-mediated Fenton reaction. Subsequently, the acidity of cancer cells treated with pLFePt-GOx was also detected using a specific pH-sensitive BCECF-AM probe whose green fluorescence weaken with the decrease of pH. As shown in Additional file 1: Fig. S9a, the green fluorescence of 4T1 cells treated with pLFePt had no significant variation, indicating that pLFePt cannot decrease cellular pH. However, pLFePt-GOx treated cells showed a significant lower green fluorescence signal compared to control and pLFePt groups. The fluorescence of 4T1 cells treated with pLFePt-GOx was also quantitatively analyzed using a fluorescence spectrophotometer, showing a similar result with CLSM observation (Additional file 1: Fig. S9b). These results confirmed that pLFePt-GOx could effectively decrease cellular acidity. Next, cellular ROS levels were also measured through the DCFH-DA probe (Fig. 3f). Compared to the saline group, the ROS levels of MDA-MB-231 cells treated with FePt, LFePt, and pLFePt were significantly increased, being the highest in the pLFePt treatment group, indicating that FePt alloys effectively activated cellular ROS production and the targeting ligand could further accelerate this process. As expected, LFePt-GOx-treated MDA-MB-231 cells showed the strongest ROS production compared to other groups, confirming excellent synergistic action of GOx and FePt alloys for activating ROS through H_2_O_2_ level and acidity enhancement. In addition, flow cytometry quantitative analysis of ROS in cells was consistent with CLSM observation (Fig. 3h).

### Cytotoxicity and anticancer mechanism of pLFePt

**Fig. 4:**
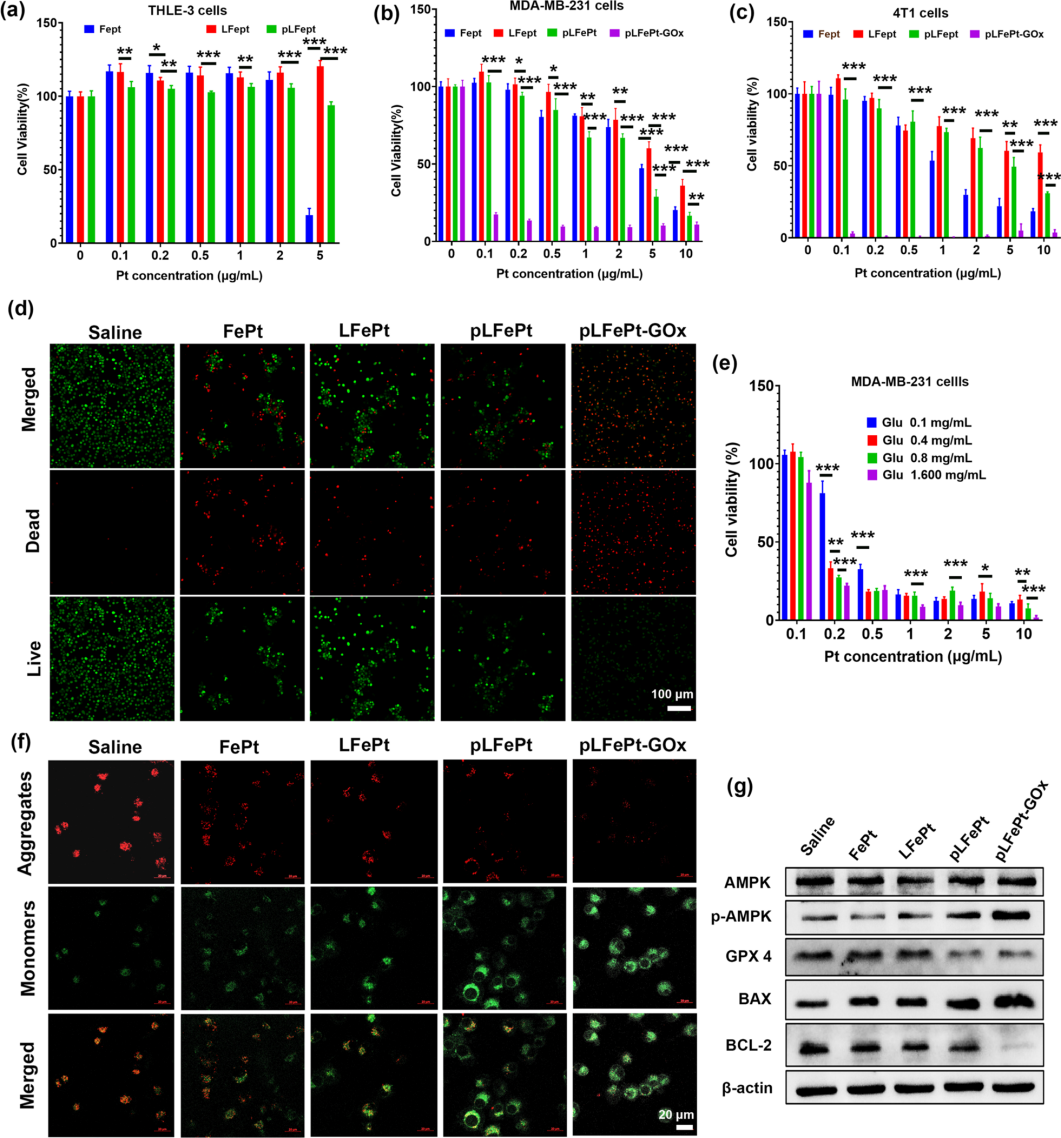
**a** The viability of THLE-3 cells treated with FePt, LFePt, and pLFePt. The viability of (b) MDA-MB-231 and **c** 4T1 cells treated with FePt, LFePt, pLFePt, and pLFePt-GOx. **d** CLSM images of Calcein-AM and propidium iodide (PI) co-stained MDA-MB-231 cells with different treatments. **e** The viability of MDA-MB-231 cells treated with pLFePt-GOx under different concentrations of glucose (n = 5, mean ± SD, *Means p < 0.05, **Means p < 0.01, ***Means p < 0.001). **f** CLSM images of MDA-MB-231 cells stained with JC-1 after treatment with different samples. **g** Western blot analysis of the protein expression of Bcl-2, Bax, GPX4, AMPK, and p-AMPK after different samples treatment. The cropped images originated from the white rectangles in Additional file 1: Fig. S11

To assess the biocompatibility of particles, normal cells (THLE-3) were incubated with FePt, LFePt, and pLFePt, and then their viability was observed through a CCK-8 assay. The viability of THLE-3 cells treated for 24 h with LFePt and pLFePt had no significant decrease at the Pt concentration range of 0.1–5 μg/mL, but at the same concentration, FePt caused the death of cells (Fig. 4a), indicating that PDGFB-targeting liposome could dramatically decrease the toxicity of ultrasmall FePt alloys. This result also indicated that pLFePt had excellent biocompatibility. In general, the cellular ROS level maintains the relative dynamic balance even during a short-team CDT treatment, which is attributed to the strong self-adjustment response of cancer cells [44]. However, with the sustained increase in cellular ROS, the balance of redox in cancer cells is broken, thus triggering cell apoptosis. Based on this, the cytotoxicity of pLFePt-GOx against cancer cells was investigated via a CCK8 assay. As shown in Fig. 4b, c, FePt alloys had a certain inhibitory effect on cell viability, which could be attributed to the functions of chemotherapy and CDT. In addition, the toxicity of pLFePt was significantly stronger than that of LFePt, which was mainly attributed to the excellent tumor targeting ability of pLFePt. Notably, pLFePt-GOx quickly induced cancer cell death even at a very low dosage (0.1 μg/mL), exhibiting the strongest anticancer activity among all treatments. Besides, we also compared cytotoxicity of pLFePt-GOx and GOx in MDA-MB-231 and 4T1 cells. The concentration of each group was standardized with GOx content. And the results showed that pLFe-Pt-GOx had more efficient toxicity than GOx in MDA-MB-231 and 4T1 cells (Additional file 1: Fig. S10). AM/PI live/dead staining analysis also confirmed that pLFePt-GOx triggered maximum cell death compared to other treatments (Fig. 4d). Together, these results demonstrate the strong synergistic effect of GOx and ultrasmall FePt alloys on the induction of cancer cell death. To further analyze the role of GOx, the viability of MDA-MB-231 cells treated with pLFePt-GOx was investigated at different glucose concentrations (Fig. 4e). The results demonstrated that the viability of cells treated with pLFePt-GOx was closely associated with the concentration of glucose, indicating that pLFePt-GOx induced cancer cell death via the synergistic action of CDT and starvation therapy. Based on the above analysis, it could be concluded that pLFePt-GOx possesses excellent anticancer activity with low toxicity.

To confirm the action of chemotherapy, the Pt–DNA adduct of cells treated with FePt, LFePt, and pLFePt were measured using ICP-MS. The result confirmed the presence of Pt–DNA adduct in 4T1 cells after FePt, LFePt, and pLFePt treatments. Furthermore, the Pt–DNA adduct level of 4T1 cells treated with pLFePt was highest, compared to FePt and LFePt, demonstrating strongest chemotherapeutic effect (Additional file 1: Fig. S12). Subsequently, the anticancer mechanism of pLFePt-GOx was also investigated through JC-1 staining and western blot analysis. Cellular ROS is mainly produced by mitochondria; thus, excessive ROS production primarily damages mitochondria, causing a decrease in the mitochondrial membrane potential (MMP). As shown in Fig. 4f, MDA-MB-231 cells treated with pLFePt-GOx showed the strongest green fluorescence and lowest red fluorescence among cells from different treatment groups, indicating that their MMP was the lowest. This result demonstrated that mitochondria-mediated oxidative injury might be an important anticancer mechanism of pLFePt-GOx. Western blot analysis indicated that pLFePt-GOx-treated cells had the highest BAX expression and lowest BCL-2 expression, which further confirmed mitochondria-mediated cell apoptosis (Fig. 4g). Excessive ROS and Fe levels can induce lipid peroxidation and result in ferroptosis. GPX4 is a pivotal regulator that maintains the balance of redox in cells through the inhibition of lipid peroxidation. Herein, GPX4 expression of pLFePt-GOx-treated cells dramatically decreased when compared with that of other groups, implying that ferroptosis is also an important mechanism of the pLFePt-GOx assembly for inducing cancer cell death. GOx-mediated glucose depletion dramatically decreases cellular ATP production, which can trigger low nutrition and starvation status of cells. Cellular AMPK expression is closely associated with ATP production and plays a key role in maintaining the glucose level in cells. The consumption of glucose and ATP can induce the phosphorylation of AMPK (p-AMPK) [45]. pLFePt-GOx-treated cells showed significantly enhanced expression of p-AMPK, suggesting the deficiency of ATP in cells. Together, these findings reveal that pLFePt-GOx effectively triggers cell death through mitochondria-mediated cell apoptosis, ferroptosis, and energetic starvation.

### Anticancer effect investigation of pLFePt-GOx in vivo

**Fig. 5:**
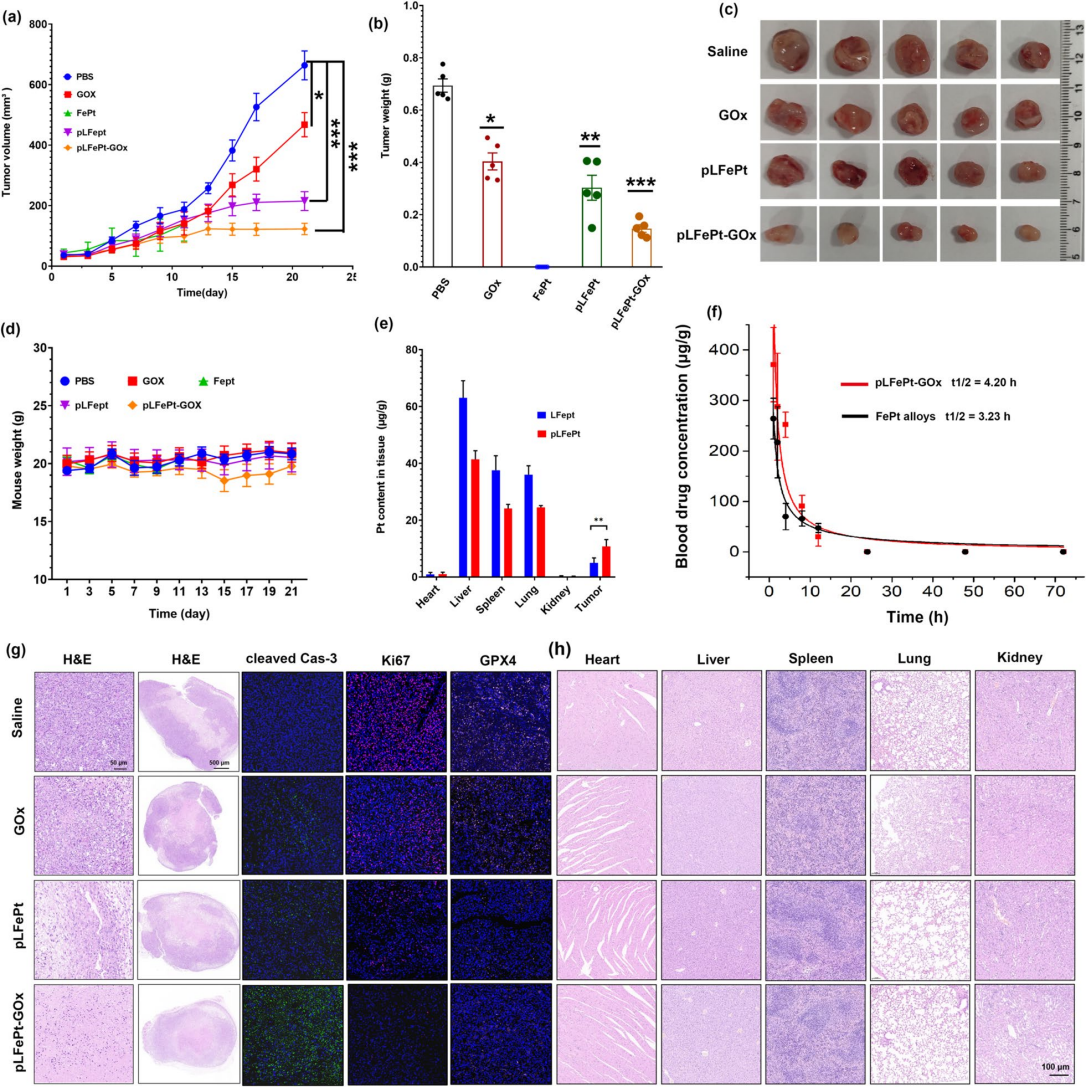
Anticancer effect of cancer-bearing mice after different treatments: **a** tumor volumes change, **b** tumor weights, **c** corresponding tumor photos. **d** Body weight change of cancer-bearing mice treated with different treatments. **e** The distribution of LFePt and pLFePt in vital organs (n = 5, mean ± SD, *Means p < 0.05, **Means p < 0.01, ***Means p < 0.001). **f** Pharmacokinetics of FePt alloys and pLFePt-GOx in mice. **g** IF and HE staining images of tumor from the mice with different treatments. **h** HE staining images of vital organs from the mice with different treatments

Based on the ROS activation ability, favorable biosafety, and excellent targeting of pLFePt-GOx, we further explored its anti-tumor activity in vivo. A breast cancer xenograft nude mouse model was constructed via subcutaneous injection of 5 × 10^6^ MDA-MB-231 cells. Mice were randomly divided into five groups that were treated with GOx, FePt, pLFePt, pLFePt-GOx, and PBS at a dosage of 2 mg/kg, respectively. Compared to PBS and GOx groups, pLFePt and pLFePt-GOx significantly inhibited the tumorigenesis of MDA-MB-231 in vivo, and the pLFePt-GOx group exhibited lowest tumor volume (Fig. 5a), confirming effective synergistic action of FePt alloy and GOx for tumor inhibition. Notably, FePt alloys caused acute death in mice; thus, no tumor volume data could be obtained. Comparatively, the mice of the pLFePt and pLFePt-GOx groups showed no death, indicating that the assembly of FePt alloys possessed better biosafety. After cancer-bearing mice were sacrificed, their tumors were exploited, weighed, and photographed. As shown in Fig. 5b, c, the weight and volume of the tumor were in the following order: PBS > GOx > pLFePt > pLFePt-GOx, which was consistent with the data in Fig. 5a. Together, these results demonstrate that pLFePt-GOx possesses the best anticancer activity. Furthermore, GOx and pLFePt groups showed no significant body weight changes compared to the control group. However, the pLFePt-GOx group a showed a slight decrease in body weight (Fig. 5d), which might be due to glucose depletion by GOx.

To confirm tumor targeting, cancer-bearing mice were administered LFePt and pLFePt at a dosage of 5 mg/kg. At 6 h post-injection, the vital organs of mice were weighed and then treated with concentrated nitric acid. Pt content in the resulting solutions were assessed using ICP-MS to analyze the bio-distribution of LFePt and pLFePt in mice. As shown in Fig. 5e, LFePt and pLFePt were mainly distributed in the liver, spleen, and lungs. Notably, the accumulation of LFePt and pLFePt in kidneys was very low, implying that LFePt and pLFePt might be excreted from the body through feces. In addition, the pharmacokinetics result indicated that the half-time of pLFePt-GOx in vivo was about 4.2 h and that it could be completely metabolized 48 h post-injection. This result demonstrated that pLFePt-GOx has a long blood circulation time and its long-term retention cannot cause toxicity to the body. Furthermore, the anti-cancer activity and biosafety of pLFePt-GOx were also investigated by pathological analysis (Fig. 5f). In addition, these tumor slices were also stained with DHE (Dihydroethidium) probe to observe ROS production. As shown in Additional file 1: Fig. S13, pLFePt-GOx group exhibited strongest red fluorescence intensity compared to other groups, indicating that pLFePt-GOx induced strongest ROS production in tumor. This tendency was consistent with the ROS staining in vitro. This result further demonstrated ROS-mediated cancer cell death induced by pLFePt-GOx. Hematoxylin and eosin (H&E) staining of tumors showed that pLFePt-GOx induced more necrotic area and abnormal nucleus phenotype, followed by pLFePt and GOx, compared to that induced by PBS (Fig. 5g). Immunofluorescence (IF) results demonstrated that the pLFePt-GOx group had the highest expression of cleaved caspase 3 and the lowest expression of Ki67 and GPX4 among all treatment groups, revealing that pLFePt-GOx treatment caused oxidative stress-induced apoptosis and lipid peroxidation, which further repressed the proliferation of tumor cells (Fig. 5g). Moreover, H&E staining of the vital organs revealed no obvious abnormal phenotype (Fig. 5h), indicating excellent tissue biosafety of pLFePt-GOx.

### In vitro and in vivo MRI evaluation

**Fig. 6:**
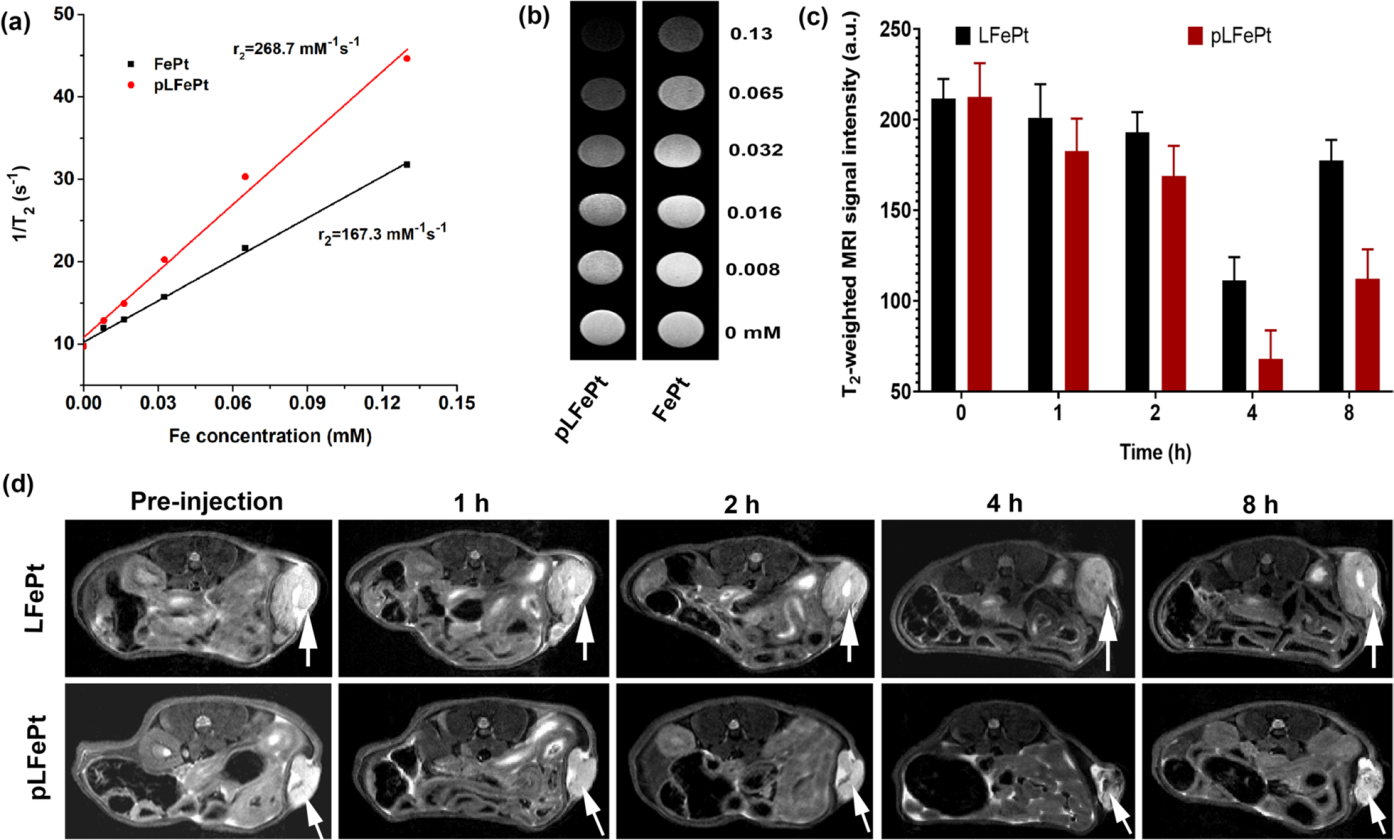
**a** T_2_ relaxation rate of FePt and pLFePt under 7.0T MRI scanner. **b** T_2_-weighted MR images of the solution containing different concentrations of FePt and pLFePt. **c** T_2_-weighted MRI signal change of tumor treated with LFePt and pLFePt. **d** T_2_-weighted MR images of tumor at axial plane at post-injection of LFePt and pLFePt

Magnetic nanoparticles commonly possess some T_2_ MRI contrast capability [46]. Considering this, we explored the possibility of pLFePt-GOx as a T_2_ MRI contrast agent. As shown in Fig. 6a, the T_2_ relaxation rate of FePt alloys reached up to 167.3 mM^−1^S^−1^, which was a relatively higher value compared to clinical feridex, implying that FePt alloys could be an ideal T_2_ contrast agent. Notably, compared to FePt alloys, pLFePt had a higher T_2_ relaxation rate (268.7 mM^−1^S^−1^), showing stronger T_2_ MRI contrast capability. It might be that the assembly of pLFePt possessed stronger magnetization, resulting in a higher T_2_ relaxation rate. The corresponding T_2_ signal images of pLFePt were also darker and showed stronger attenuation than that of FePt alloys, further confirming that pLFePt had better T_2_ MRI contrast performance than FePt alloys (Fig. 6b). The excellent T_2_ relaxation rate of pLFePt encouraged us to further explore its tumor diagnosing ability in vivo. For this, cancer-bearing mice were intravenously administrated FePt alloys and pLFePt at a dosage of 5 mg/kg. As can be observed in Fig. 6d, the T_2_-weighted MRI images of tumors injected with LFePt and pLFePt was significantly darker compared to pre-injection, and the darkest tumor images appeared at 4 h post-injection. In addition, the MR images of tumors treated with pLFePt were dramatically darker than that of tumors treated with LFePt, implying that pLFePt had a better tumor diagnosing ability. This was because pLFePt with excellent tumor targeting promoted the accumulation of particles in the tumor, thus achieving more MRI contrast enhancement. Subsequently, the signals of the tumor region in MR images were analyzed through MRIcro software (Fig. 6c). The results indicated that the MRI signal intensity of tumor treated with LFePt and pLFePt significantly shortened, and the lowest MRI signal intensity appeared at 4 h post-injection. The results were similar to that of MR images of tumors. The MRI signal and brightness of the tumor significantly recovered at 8 h post-injection, implying that pLFePt began to gradually undergo metabolization in the tumor. Taken together, these results revealed that pLFePt exhibits excellent tumor targeting activity and stronger T_2_ MRI contrast ability, which would be beneficial for accurate theranostic of tumors.

## Conclusion

In summary, a specific tumor targeting, TME responsive nanotheranostic agent (pLFePt-GOx) has been successfully fabricated for starvation-enhancing CDT of cancer. The pLFePt-GOx showed an excellent tumor targeting ability and ultrahigh Fenton-catalytic activity. These characteristics make it a promising agent for the accurate CDT treatment of carcinomas. Upon entry into cancer cells, the pLFePt-GOx was degraded owing to the weakly acidic nature of TME, thus releasing large amounts of FePt alloys and GOx. The released GOx-mediated glucose consumption not only caused a starvation status but also increased the cellular H_2_O_2_ level and acidity to promote Fenton reaction by FePt alloys, increasing ROS accumulation in the cells, which ultimately realized starving-enhanced CDT for cancer. In addition, cellular high oxidative stress and acidity also triggered the release of a large amount of Pt(II) ions from FePt alloys, contributing to cancer chemotherapy. Besides, pLFePt-GOx showed excellent T_2_ relaxivity and its systemic delivery significantly enhanced the MRI contrast signal of tumor, acquiring high quality of tumor MR images and promoting tumor accurate diagnosis. Therefore, this study provides information on a novel Fe-based theranostic platform, which can be applied for MRI-guided starvation-enhancing CDT of cancer.

## Supplementary Information

**Additional file 1: Fig. S1.** The photo of FePt alloys solution. **Fig. S2.** The high-magnification TEM images of LFePt-GOx. **Fig. S3.** ESR spectra. **Fig. S4.** The degradation rate of MB solution. **Fig. S5.** Hydrodynamic size change of pLFePt-GOx. **Fig. S6.** XPS spectra of pLFePt. **Fig. S7.** The pH variation of pLFePt-GOx solution**. Fig. S8.** Pt release profile of pLFePt. **Fig. S9.** Cellular pH analysis. **Fig. S10. Cytotoxicity of** GOx and pLFePt-GOx. **Fig. S11.** The original images of western blot analysis. **Fig. S12.** Pt–DNA adduct content of 4T1 cells after different samples treatment. **Fig. S13.** In vivo ROS-staining images of the tumors.

## Data Availability

The datasets and materials used in the study are available from the corresponding author.
