# Supplementary material for: PDGFB targeting biodegradable FePt alloy assembly for MRI guided starvation-enhancing chemodynamic therapy of cancer
Source: J Nanobiotechnology. 2022 Jun 7;20:264. doi: 10.1186/s12951-022-01482-x (PMC9172083; doi:10.1186/s12951-022-01482-x)
Supplement: Supplementary file 1 — Additional file 1: Fig. S1. The photo of FePt alloys solution. Fig. S2. The high-magnification TEM images of LFePt-GOx. Fig. S3. ESR spectra. Fig. S4. The degradation rate of MB solution. Fig. S5. Hydrodynamic size change of pLFePt-GOx. Fig. S6. XPS spectra of pLFePt. Fig. S7. The pH variation of pLFePt-GOx solution. Fig. S8. Pt release profile of pLFePt. Fig. S9. Cellular pH analysis. Fig. S10. Cytotoxicity of GOx and pLFePt-GOx. Fig. S11. The original images of western blot analysis. Fig. S12. Pt–DNA adduct content of 4T1 cells after different samples treatment. Fig. S13. In vivo ROS-staining images of the tumors. [file 12951_2022_1482_MOESM1_ESM.doc]

**Supplementary Material**

**PDGFB targeting biodegradable FePt alloy assembly for MRI guided starvation-enhancing chemodynamic therapy of cancer**

Caiyun Zhang^1^, Zhiguo Leng^1^, Yinfeng Wang, Lang Ran, Xia Qin, Huan Xin, Xiaotong Xu, Guilong Zhang*, and Zhaowei Xu*

School of Pharmacy, Shandong Technology Innovation Center of Molecular Targeting and Intelligent Diagnosis and Treatment, Binzhou Medical University, Yantai, 264003, P. R. China

*Corresponding author.

Email: zhaoweixv@bzmc.edu.cn; glzhang@bzmc.edu.cn.

^1^ Co-first author.

**Experimental section**

**Reagents and antibodies.** Platinum Bis(Acetylacetonato) (C_10_H_14_O_4_Pt，98%), 1,2-Hexadecanediol (C_16_H_34_O_2_, 98%) were acquired from Shanghai Yuanye Bio-Technology Ltd (China). Nonacarbonyldiiron (C_9_Fe_2_O_9_,97%), Glucose Oxidase (9001-37-0), meso-2,3-Dimercaptosuccinic acid (DMSA), and fluorescein isothiocyanate (FITC), Chlorpromazine hydrochloride (69-09-0) were obtained from Aladdin Reagents Co. Ltd (China). DSPE-MPEG2000 (F01008), HSPC-90A (N01003), CHO-HP (57-88-5) were purchased from A.V.T. Pharmaceutical Co. Ltd (China). mPEG-NH_2_ (2000) was acquired from Macklin (China). Trimethylbenzene (TMB, BR), BCEC AM (S1006) were obtained from Sangon Biotech Co. Ltd (China). Cell Counting Kit-8 (CCK-8) (B34302), containing protease inhibitor cocktail (B1400) were obtained from Biomarker technologies CO.,LTD. Mitochondrial membrane potential assay kit with JC-1 (C2006) and Reactive Oxygen Species Assay Kit (DCFH-DA) (S0033S), Hydrogen Peroxide Assay Kit (S0038), WB/IP lysis buffer (P0013), BCA protein assay kit (P0011) were purchased from Beyotime Biotechnology Co., Ltd. DNA Extraction kit (AG21009) was obtianed from Accurate Biology. ROSGreen H_2_O_2_ Probe (MX5202-1MG) was purchased from Shanghai Mao Kang biotechnology Co,Ltd. Amiloride HCl (17440-83-4), Living/Dead cell double staining kit (Calcein-AM/PI), Hoechst 33342 (C0031) were acquired from Beijing Solarbio Science & Technology Co.,Ltd. Dihydroethidium (DHE) (YT301) was obtained from Beijing Baiaolaibo Technology Co., Ltd. Antibodies against GPX4 (DF6701), BCL-2 (AF6139), Caspase-3 (DF6020) were acquired from Affinity Biotech Co. Ltd (USA). BAX (5023S), AMPK (2532S), P-AMPK (2535S) antibodies were obtained from Cell Signaling Technology Co. Ltd (USA). Some other chemical agents, if not mentioned, were commercially obtained from Sinopharm Group Chemical Reagent Co., Ltd.

**Synthesis of** **hydrophilic FePt alloy.** Typically, 0.1 mg of Pt(acac)_2_ and 0.2 mg of 1,2-hexadecanediol were added into a three-neck flask containing 20 mL of dibenzyl ether. The mixed solution was heated to 100 °C under magnetic stiring for 30 min, and then 160 μL of oleic acid, 170 μL of oleylamine and 0.3653g of nonacarbonyldiiron were further added into the reaction system. Next, the solution was further heated to 300 °C under reflux with nitrogen gas flow for 3 h. Finally, the reaction solution was naturally cooled to room temperature and then washed with ethanol twice, the product was dispersed into the [n-hexane](../../../../D:/%25E6%259C%2589%25E9%2581%2593%25E8%25AF%258D%25E5%2585%25B8/Dict/8.10.3.0/resultui/html/index.html#/javascript:;) solution for further characteristic analysis.

Firstly, 5 mL of DMF solution containing NOBF_4_ (0.1 mM) was added into the FePt n-hexane solution, then the mixture was sonicated for 2 min. When the solution was stratified and the supernatant mainly containing n-hexane was discarded. Secondly, 0.05 g of DMSA was further added into the FePt DMF solution, then the mixed solution was rocked at 170 rpm for 40 min at 37°C. After vibration, the generated FePt-DMSA was centrifuged at 14000 rpm for 10 min and the obtained products were then washed with deionized water and ethanol at least for three times.

**Synthesis of pLFePt-GOx.** For preparation of PDGFB-PEG, 5 μg of PDGFB, 100 mg of NHS and EDC were dissolved into 5 mL of [dimethyl](../../../../D:/%25E6%259C%2589%25E9%2581%2593%25E8%25AF%258D%25E5%2585%25B8/Dict/8.10.3.0/resultui/html/index.html#/javascript:;) [sulfoxide](../../../../D:/%25E6%259C%2589%25E9%2581%2593%25E8%25AF%258D%25E5%2585%25B8/Dict/8.10.3.0/resultui/html/index.html#/javascript:;) (DMF) solution. Then, the mixture was stirred for 2 hours and the 10 mg of PEG-NH_2_ (M.W.2000) was quickly added into the reaction system. After stirring for 6 hours, the resulting PDGFB-PEG was dialyzed using a 1500 KDa dialysis bag for three times to remove the DMF, and the PDGFB-PEG was collected in the aqueous phase. After that, 500 μg of PDGFB-PEG was dispersed into chloroform，1 mL of HSPC (10 mg/mL), 60 μL of HEPC (10 mg/mL) and 200 μL of DSPE (10 mg/mL) were then added into the mixture. Then, the mixed solution was treated to remove solvent and form a film using a rotary evaporator at 37 °C. Next, 5mg of GOx dissolved in 10 mL deionized aqueous solution was added to the bottle and was continuously rotated for 3 min. Afterwards, the resulting solution was sonicated for 10 min, then the FePt solution was further added and sonicated for 20 min. Finally, the mixture was centrifuged at 10000 rpm for 10 min and the sediment was dispersed into deionized water.

**Measurement for the catalytic activity of LFePt in solution.** To measure ·OH generation by ESR spectroscopy, the FePt alloys, MIO, and USIO ( pH 4.5, 1 mL, 20 mM) were treated with 30 μL of H_2_O_2_ (30 wt %) and 100 μL (200 mM) of 5,5-dimethyl-1-pyrroline-N-oxide (DMPO), respectively. After treatment for 20s, these samples were measured by ESR spectroscopy. In addition, the ·OH generation was also detected with colorimetric methylene blue (MB) assay. In detail, different concentrations of pLFePt was added into 10 mM of glucose solution containing 2% of H_2_O_2_ and 0.5mM of MB at pH 7.4, 6.5, 5.5, and 4.5. After incubation for 12 h, the absorption of MB was recorded using a UV-Vis spectrometer with 650 nm of wavelength.

**Release behavior of Pt from pLFePt.** 5 mg of LFePt was uniformly dispersed into 4 mL of PBS with and without 10 μM of H_2_O_2_ at pH 4.5, and then the mixed solution was incubated in a shaker for 48 h at 37 °C. Subsequently, the solution was centrifuged at 14000 rpm at different time intervals, and the supernatant liquid was collected. Finally, Pt content in the supernatant liquid was measured using ICP-OES. The release rate (RA) of Pt was calculated according to the following equation:

RA(%) = C_Pt_ × V/(M_nano_ × LC) × 100 % (1)

where C_Fe_ was the Pt concentrations of the supernatant solutions, V was the total volume of the solution, M_nano_ was the mass of pLFePt, and LC was the ratio of Pt in pLFePt.

**Cell Culture.** MDA-MB-231 cells were cultured in Dulbecco modified Eagle medium (DMEM, SH30081, Hyclone) containing 10% fetal bovine serum (FBS, 10099141C, Gibco), 100 μg/mL penicillin and 100 μg/mL streptomycin. THLE-3 cells were maintained in RPMI-1640 (SH30605, Hyclone) supplemented with 10% FBS, 100 μg/mL penicillin and 100 μg/mL streptomycin. 4T1 cells were cultured in RPMI-1640 complete medium with 10% fetal bovine serum, 100 μg/mL penicillin and 100 μg/mL streptomycin. All cells were cultured in the HERAcell incubator at 37 °C and 5% CO_2_.

**Extracellular and cellular H_2_O_2_ detection.** The H_2_O_2_ generation induced by GOx-mediated glucose consumption was investigated using the Hydrogen Peroxide Assay Kit. For the extracellular H_2_O_2_ detection, the LFePt-GOx containing 50 μg/mL GOx were incubated in a reaction buffer with different glucose concentrations. After incubation for 6 h, the mixture was centrifuged at 14000 rpm for 10 min, then the supernatant liquid were collected for the detection of H_2_O_2_ content according to the manufacturer’s instruction. For cellular H_2_O_2_ detection, MDA-MB-231 cells were pretreated with FePt, LFePt, pLFePt or pLFePt-GOx (Fe: 5 μg/mL) for 4h, then the cellular H_2_O_2_ were evaluated with Hydrogen Peroxide Assay Kit (BC3595, Solarbio Biotechnology) which reacted with H_2_O_2_ to generate yellow titanium superoxide with significant absorbance of 415 nm or staining with ROSGreen^TM^ H_2_O_2_ probe Kit (MX5202, Maokang Biotech) according to the manufacturer‘s protocol. The cellular content of H_2_O_2_ in MDA-MB-231 were measured or collected with spectrophotometer or confocal laser scanning microscope (CLSM).

**Extracellular and cellular pH detection.** The pLFePt-GOx (GOx: 50 μg/mL) was incubated in the reaction buffers with the range of 0-12.5 mM of glucose at 37 °C. After incubation for 12 h, the pH value of the solutions were measured with a pH meter. Subsequently, the pH changes of MDA-MB-231 cells treated with pLFePt and pLFeP-GOx (10 μg/mL) were observed using a BCECF-AM pH fluorescent probe by confocal laser scanning microscope. After incubation for 6 h, the cells were stained with BCECF-AM (2.5 μM) for 30 min, and a nuclear staining reagent Hoechst 33342 (1 μg/mL) for 30 min in dark room. After washing three times with PBS, the cells were observed by CLSM. Moreover, the intracellular fluorescent intensity was also detected by fluorescence spectrophotometer.

**In vitro and in vivo MRI investigation.** For *in vitro* MR assessment, a set of spin echo images of different concentrations of FePt and pLFePt were collected to achieve their transverse relaxation time (T2). The detailed parameters were as follows: (TR 6000 ms, effective TE 5.6 ms, BW 25 kHz, slice thickness 1 mm, matrix 128×128, 1 average) except for the 20 different inversion times (TIs) that were varied linearly from 10 to 2500 ms. In addition, T_2_-weighted MR images of the tumor were acquired at different times with axial orientations using a spin-echo sequence. The detailed parameters were as follows: repetition time (TR) = 370 ms, echo time (TE) = 11.6 ms, field of view (FOV) = 35 mm × 35 mm, matrix size = 256 × 256, slice thickness = 0.8 mm (16 slices, gap = 0), 1 average, and bandwidth (BW) = 50 kHz.

**Cell toxicology**. The cells’ viability was assessed by the CCK-8 kit (B34302, Biomake) according to the manufacturer’s manual. Briefly, the 4T1 and MDA-MB-231 cells at a density of 1×10^4^ cells/well were incubated with various concentrations of FePt, LFePt, pLFePt, pLFeP-GOx for 24 h. Meanwhile, the MDA-MB-231 cells were also treated with pLFeP-GOx at different concentrations of glucose (100, 400, 800, 1600 μg/mL) for 24 h. Subsequently, the culture media were removed and cells were washed with PBS for three times. Then 100 µL of fresh culture medium containing 10% CCK-8 solution was added to each well and the plate were incubated at 37 °C for 2 h. The absorbance at 450 nm was detected by a microplate reader.

**Cellular biocompatibility.** THLE-3 and MDA-MB-231 cells were seeded in 96-well plates at a density of 1×10^4^ cells per well and incubated with different concentrations of FePt, LFePt, pLFePt quantified with Pt (0.1, 0.2, 0.5, 1, 2, 5, 10 µg/mL) for 24 h. Subsequently, the culture media were removed and the cells were washed with fresh PBS for three times. Then 100 µL of fresh medium containing 10% CCK-8 solution was added to each well, and the plate were incubated at 37 °C for 2 h. The absorbance at 450 nm was detected by a microplate reader.

**Cellular ROS detection.** The cellular ROS production was evaluated by a DCFH-DA kit according to the manufacturer’s instruction. Briefly, MDA-MB-231 cells were seeded at 2×10^6^ cells/well in 6 well plates and treated with saline, FePt, pLFePt, pLFePt-GOx at a certain concentration of 5 µg/mL for 4 h. Then, the cells were collected and incubated with DCFH-DA probe (10 µM) at 37 °C for 20 min. Subsequently, the cells were collected and washed with DMEM basic medium for three times to remove the non-specific DCFH-DA probes. The cells was observed by CLSM or quantified by the flow cytometry.

**Cellular uptake** **evaluation**. The green fluorescence molecules, fluorescein isothiocyanate (FITC), were rationally labeled onto LFePt and pLFePt to investigate the cellular uptake efficiency. MDA-MB-231 cells were seeded in 6-well plates at a density of 2×10^6^ cells per well and treated with pLFePt at different concentrations for different time. Meanwhile, MDA-MB-231 cells were also treated with LFePt, pLFePt, and pLFePt with treatment of chlorpromazine, Amiloride-HCl or 4°C as indicated. Then, the nuclei were stained with Hoechst 33342 in dark conditions for 20 min. Finally, the images were collected using a CLSM. Then, the fluorescence intensity of cells was quantitatively measured by flow cytometry (CytoFLEX Beckman-Coulter, USA) and ICP-OES.

**Mitochondrial membrane potential (MMP) assay.** Mitochondrial membrane potential assay kit with JC-1 was used to detect the MMP change of MDA-MB-231 cell after treatment with 5 µg/mL of FePt, LFePt, pLFePt, or pLFePt-GOx for 4 h. Then, the cells were washed twice with PBS, and then stained with JC-1 (5 µg/mL) for 20 min. Finally, the images were captured by CLSM.

**Western Blot.** MDA-MB-231 cells were seeded in 6 cm^2^ dish at a density of 3×10^6^ cells per well. Then, the cells were treated with Saline, FePt, LFePt, pLFePt and pLFePt-GOx for 4 h. Then cells were harvested and lysed with WB/IP lysis buffer containing protease inhibitor cocktail at 4°C for 30 min. Then, the samples were centrifuged at 12000 rpm/min for 10 min, the supernatant was collected and the protein density was measured with BCA protein assay kit. 40 μg of proteins were loaded and subjected to SDS-PAGE. The proteins were transferred on PVDF-membrane and were incubated with the following primary antibodies against GPX4, BCL-2, BAX, AMPK, P-AMPK, or Caspase 3 overnight at 4 °C, and they were further incubated with the corresponding secondary antibodies at room temperature for 1 h. Finally, the protein bands were visualized using ECL imaging system.

**Live/dead cell staining.** MDA-MB-231 cells at a density of 2 × 10^6^ cells/well were treated with 10 µg/mL of FePt, LFePt, pLFePt, or pLFePt-GOx nanoparticles for 24 h respectively, and the cells treated with saline as control. Subsequently, the cells were stained with Calcein-AM/PI live/dead staining assay Kit according to the manufacturer’s manual and the cellular fluorescence was monitored by CLSM.

**Measurement of Pt-DNA adduct formation.** For the measurement of Pt-DNA adduct in MDA-MB-231 cells, the cells at a density of 5×10^5^ cells/well were cultured with saline, FePt, LFePt or pLFePt for 6 h. Total cellular DNA was purified and isolated by using SteadyPure Universal Genomic DNA Extraction kit (AG21009, Accurate Biology), and Pt content in DNA was analyzed using ICP-OES.

**Biodistribution of LFePt and pLFePt *in vivo*.** MDA-MB-231 tumor-bearing mice were intravenously injected with 10 mg/kg of pFePt and pLFePt. At 6 h post injection, the primary organs were removed, weighed, homogenized, and then nitrated using a microwave digestion system. After that, the obtained solutions were filtrated using a 0.25 µm membrane, and then the supernatant was collected and subjected to ICP-OES. The LFePt and pLFePt distribution in different organs was calculated by normalizing with Pt content and taken out the amount of Pt from the tissues of mice.

***In vivo* antitumor and biosafety evaluation.** All animal experiments and immunohistochemical analysis were approved by the Ethics Committee for Biological and Medical Science of Binzhou Medical University. Four weeks female athymic immune-deficient nude mice (BALB/c mice) were obtained from GemPharmatech Co Ltd (Nanjing, China). Animals were randomly assigned to different groups as indicated and each group had five animals. After that, 100 µL of serum-free DMEM containing 5×10^6^ of MDA-MB-231 cells were injected subcutaneously into the flank of nude mice. When tumors were 50 mm^3^, the mice were intravenously injected with saline, FePt, LFePt, pLFePt, pLFePt-GOx at a dose of 5mg/kg every two days for a total of 11 times. Meanwhile, the tumor volume and weight of tumor-bearing mice were recorded every 2 days and the tumor volumes were calculated based on V=0.5×*L*×*W*^2^ equation (*L* indicates the longest diameter and *W* is the shortest diameter). After the last treatment, mice were sacrificed, tumor and major tissues including heart, liver, spleen, lungs, and kidneys were collected, photographed and subjected to subsequent pathological analysis. The sections of tumors and organs with different treatments were stained with GPX4, Ki67 and Caspase-3 antibodies to evaluate the anti-tumor effect, the haematoxylin and eosin (H&E) staining were performed to evaluate the biocompatibility of nanoparticles. These sections were photographed by fluorescence microscope or CLSM.

**ROS staining of tumor tissue by DHE probe.** 4T1 tumor-bearing mice were intravenously injected with saline, FePt, LFePt, pLFePt, and pLFePt-GOx at a dose of 5mg/kg. Subsequently, the tumors were dissected 6 hours after the second injection and frozen sections of tumors were made for subsequent staining. The DHE working solution was prepared and diluted with double distilled water according to the protocol, and frozen sections were staining with working buffer and incubated at 37 °C for 40 min. Finally, the cellular fluorescence was monitored by CLSM.


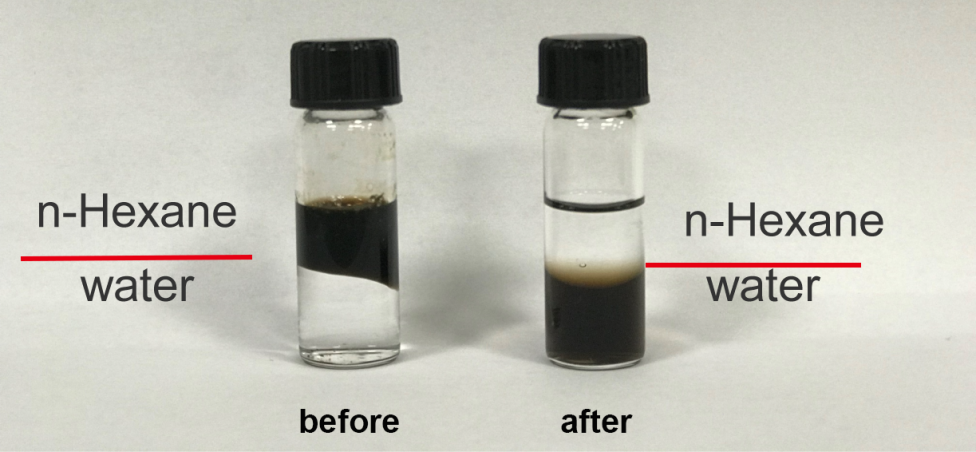


Fig S1. The photo of FePt alloys solution before and after ligand exchange.


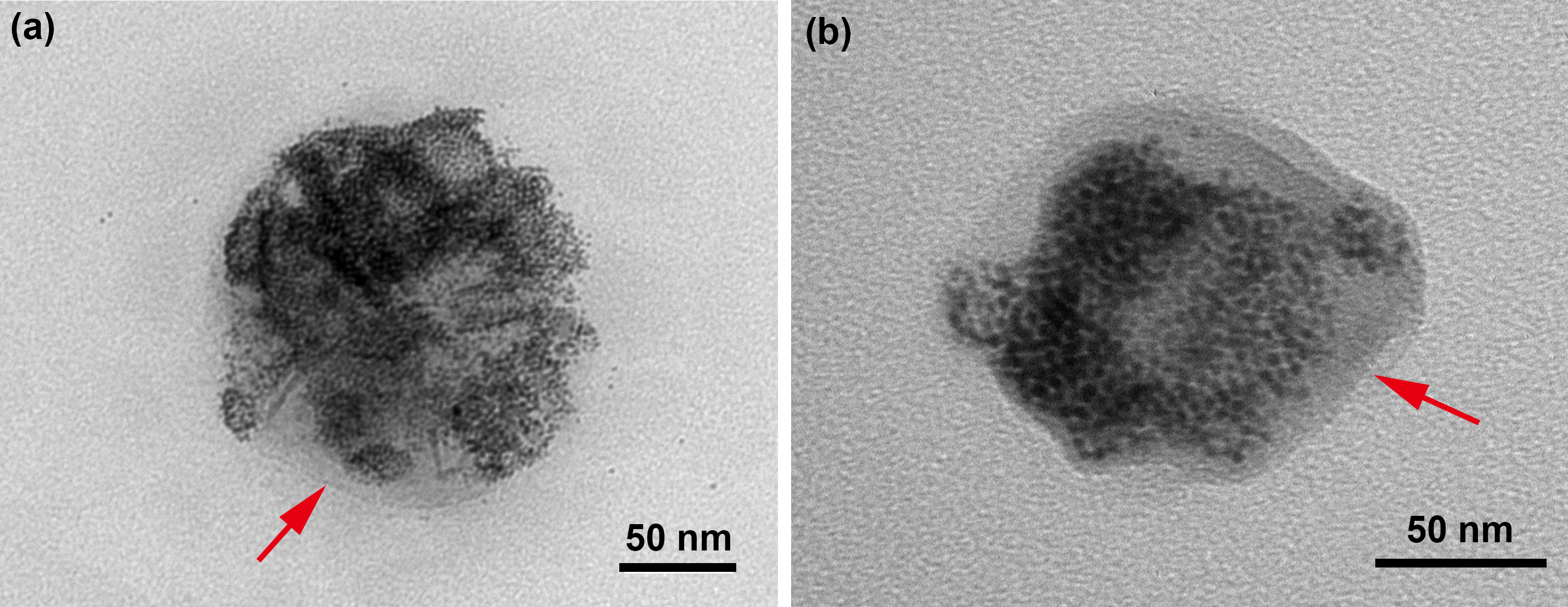


Fig S2. (a,b) The high-magnification TEM images of LFePt-GOx.


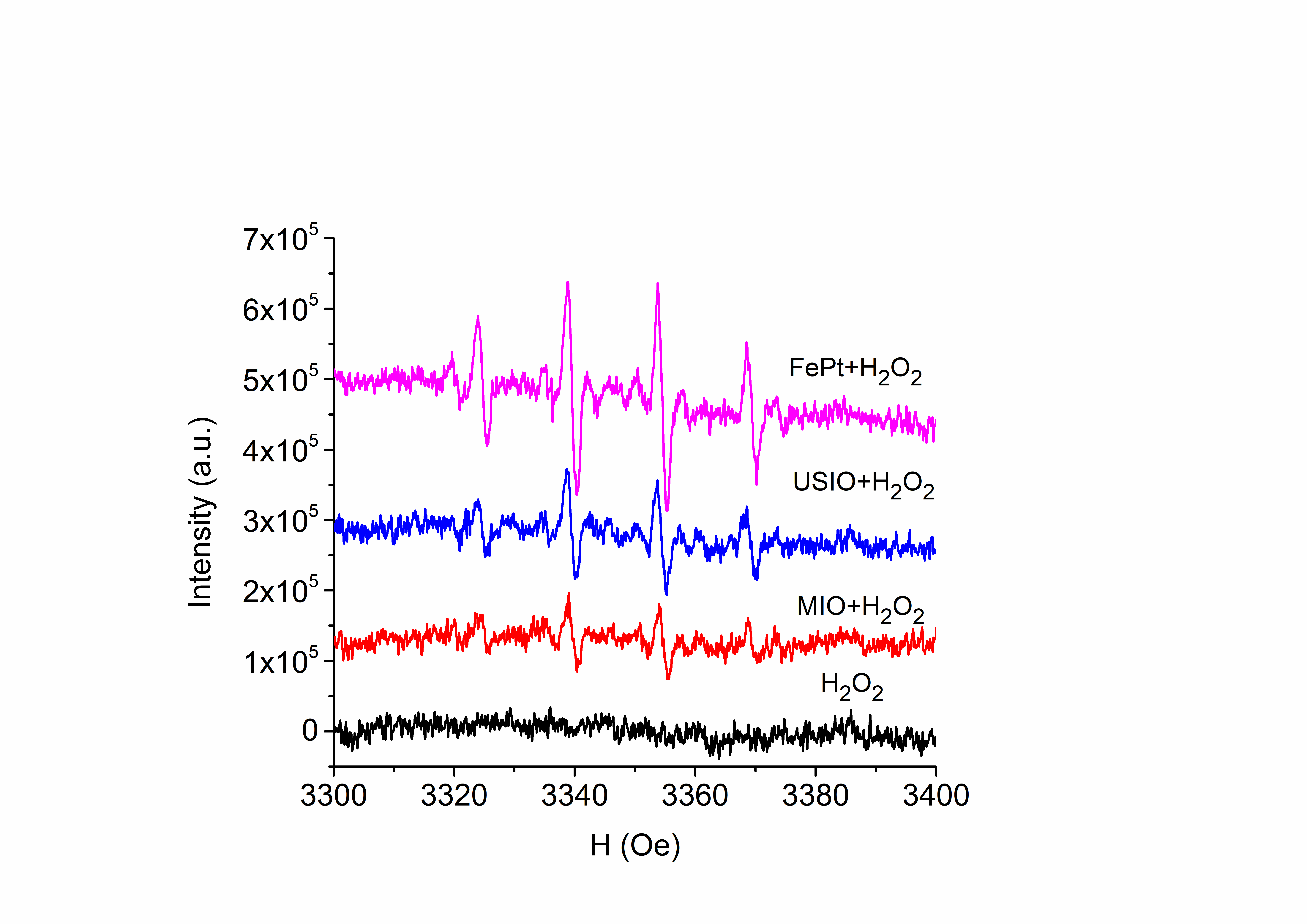


Fig S3. ESR spectra of H_2_O_2_ solution treated with MIO, USIO, and FePt alloys at pH 5.5.


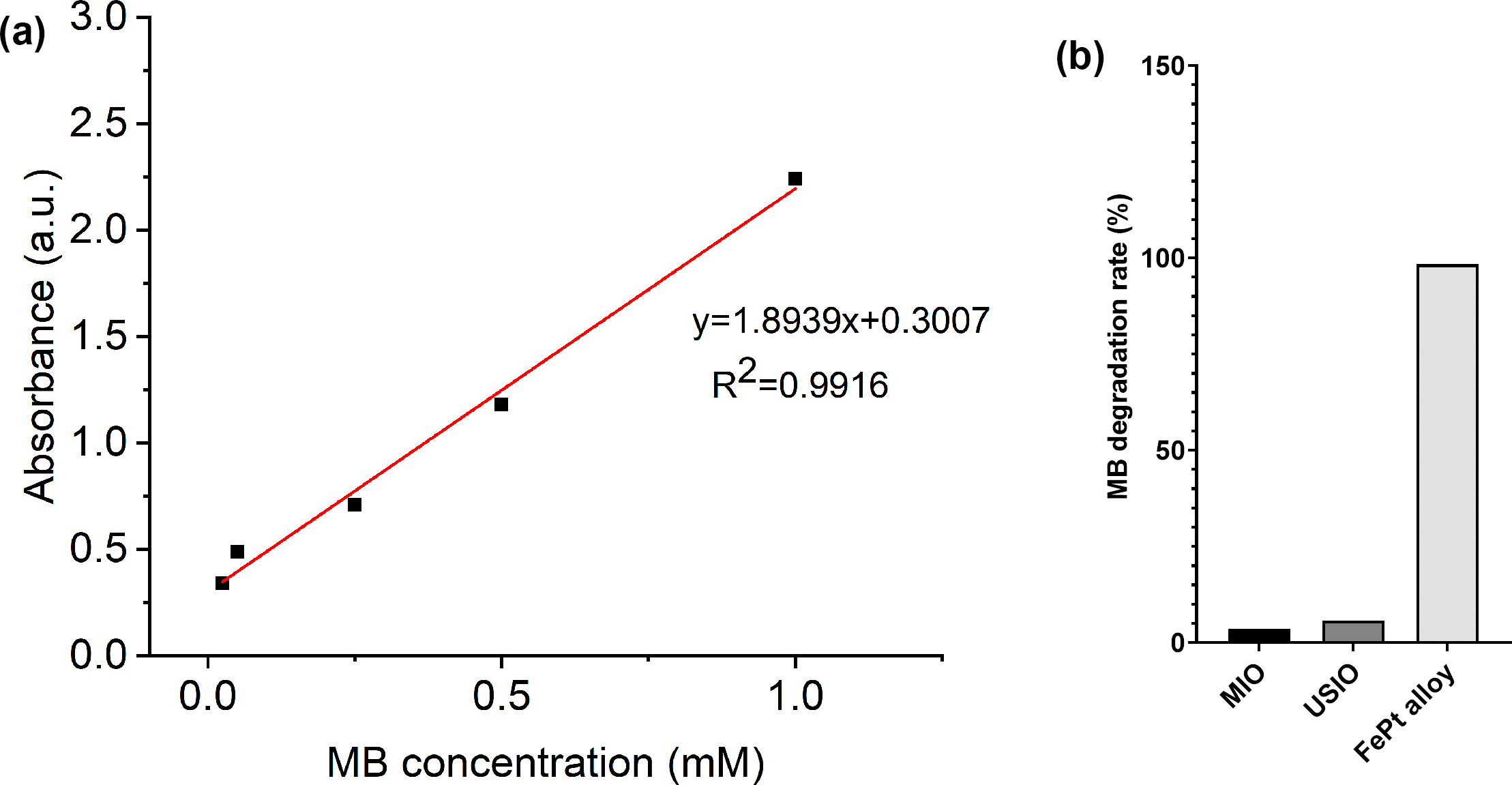


Fig S4. (a) The standard curve of MB; (b) The degradation rate of MB solution treated with MIO, USIO, and FePt alloy.


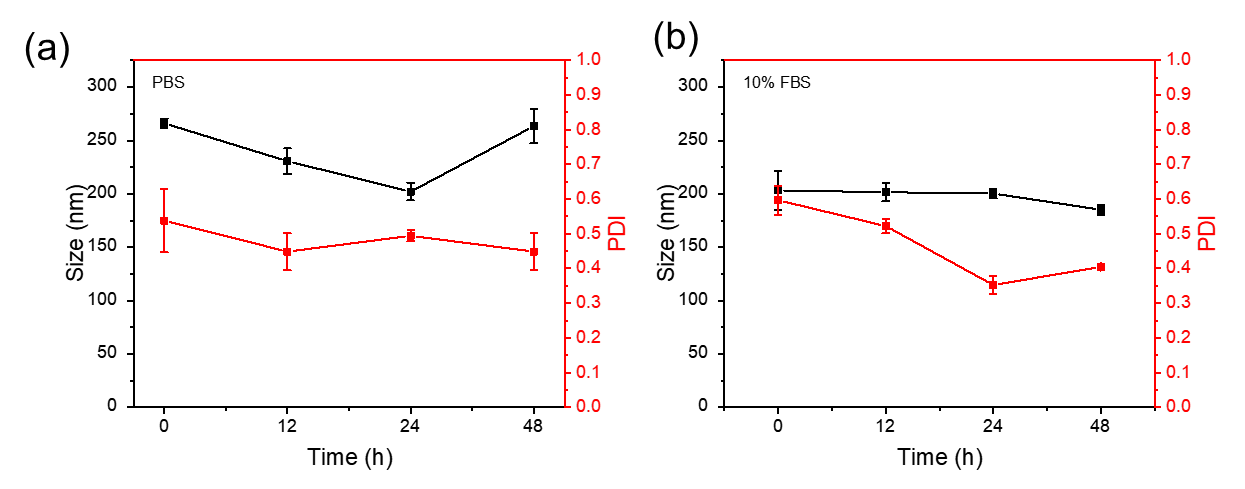
Fig S5. Hydrodynamic size change of pLFePt-GOx under (a) phosphate buffer saline (PBS) and (b) fetal bovine serum (FBS).


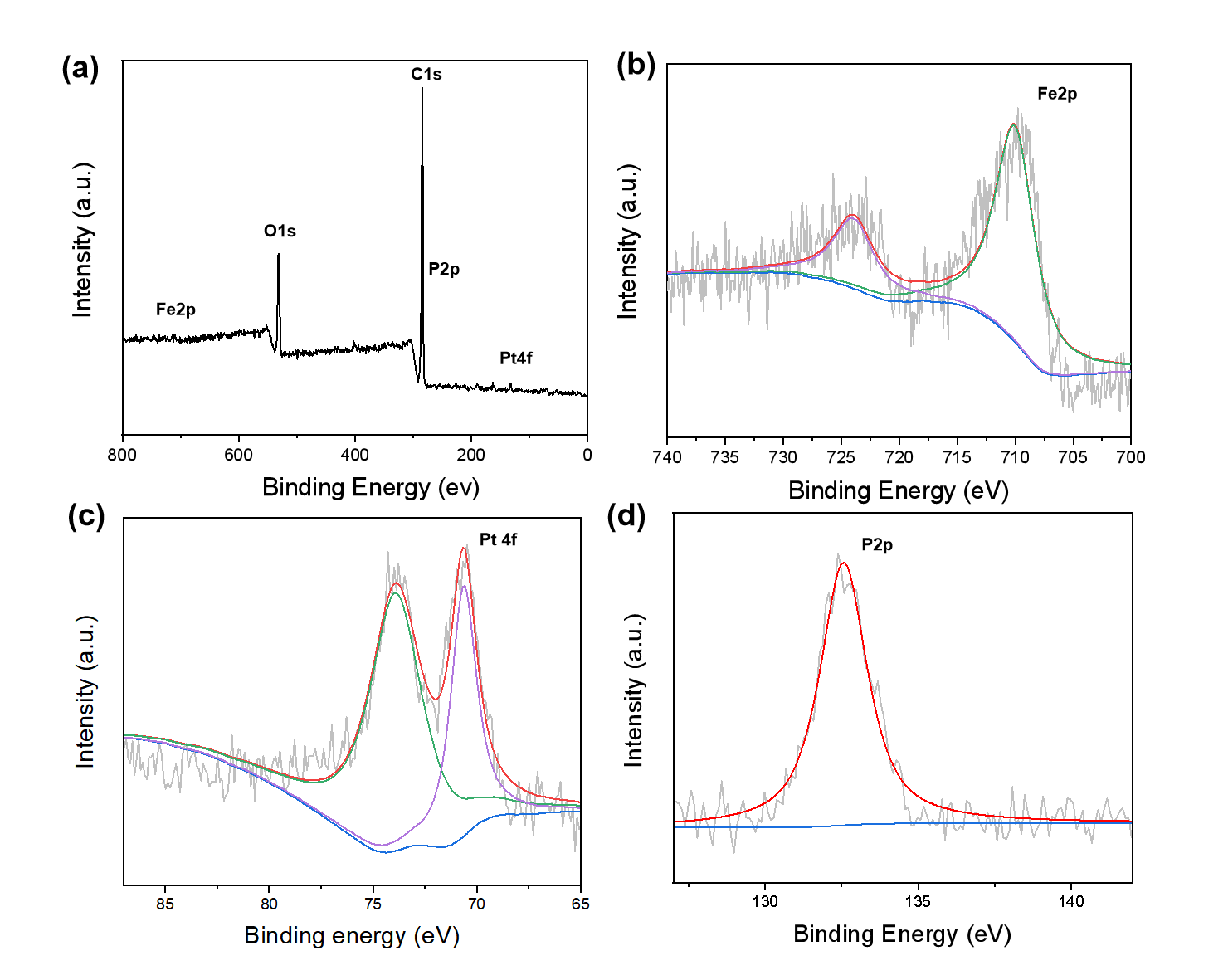


Fig S6. (a) XPS full spectra, (b) Fe2p, (c) Pt4f, and (d) P2p spectra of pLFePt.

Fig S7. The pH of pLFePt-GOx solution incubated in different concentrations of glucose for 12h.


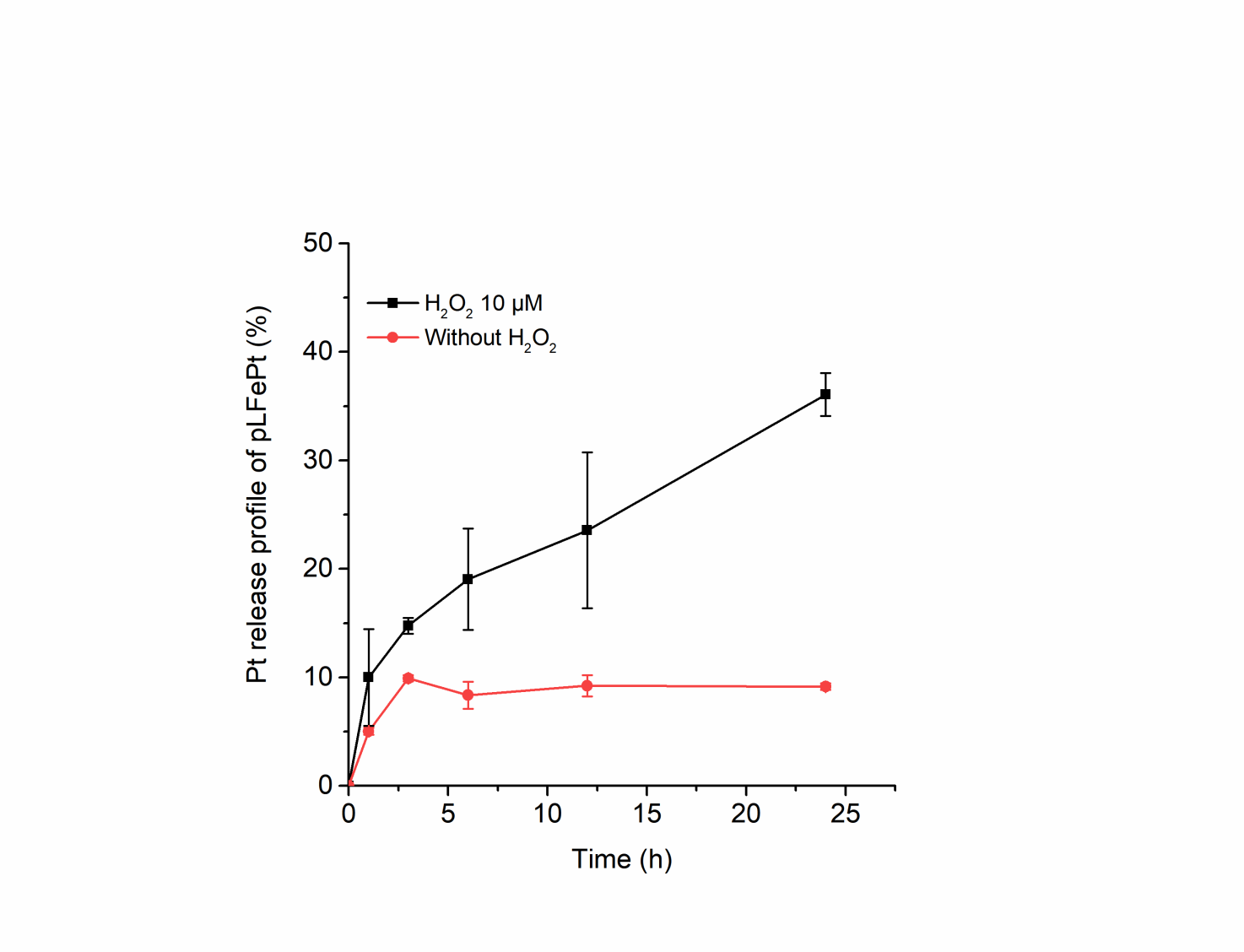


Fig S8. Pt release profile from pLFePt under weakly acidic solution (pH 4.5) with or without 10 μM H_2_O_2_.


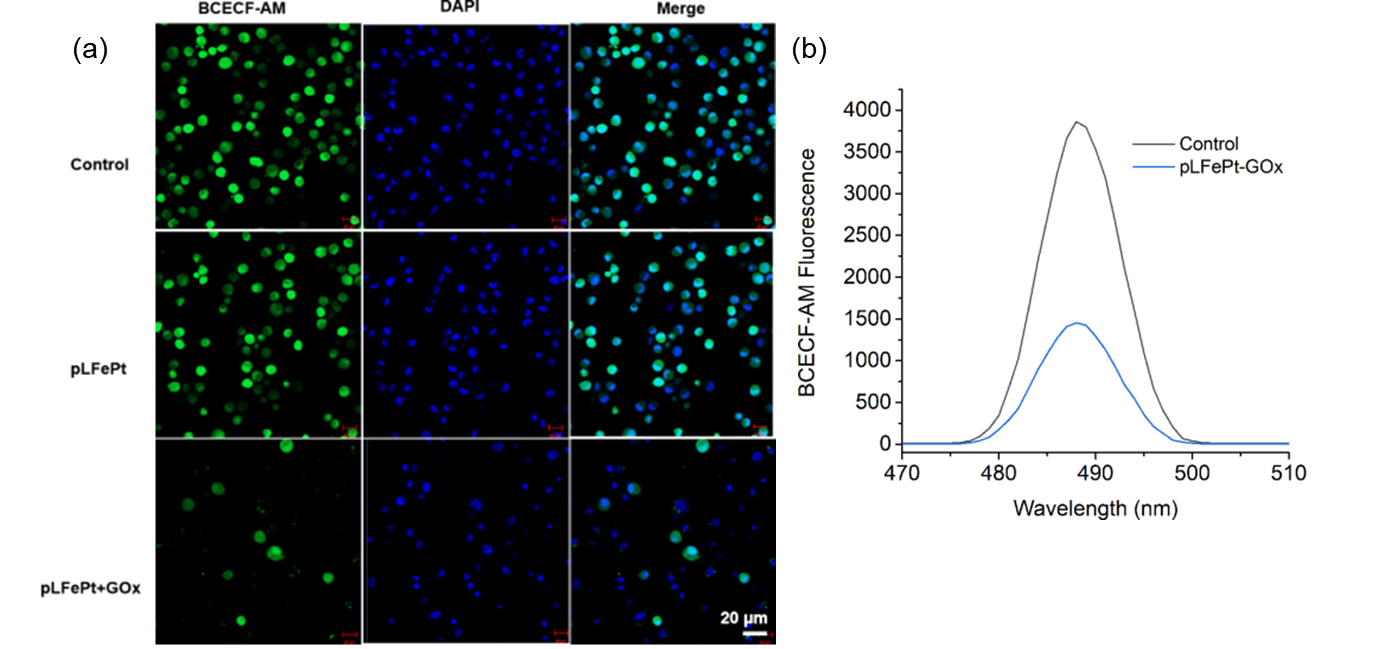


Fig S9. The (a) CLMS observation, (b) fluorescence spectrophotometry analysis for cellular pH.


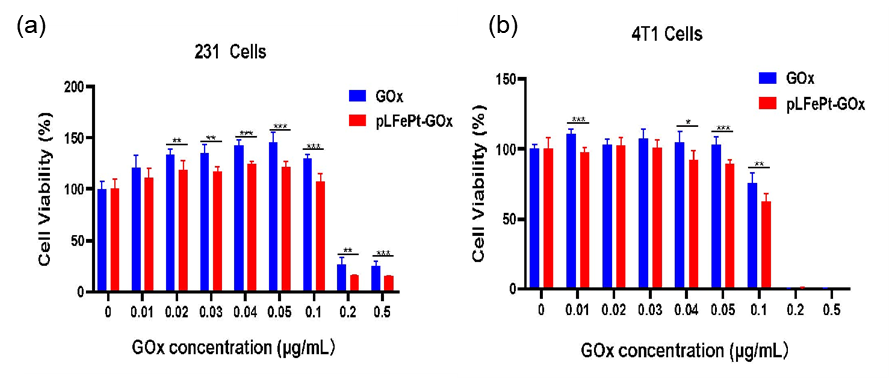


Fig S10. The viability of (a) MDA-MB-231 and (b) 4T1 cellells treated with GOx and pLFePt-GOx .

**
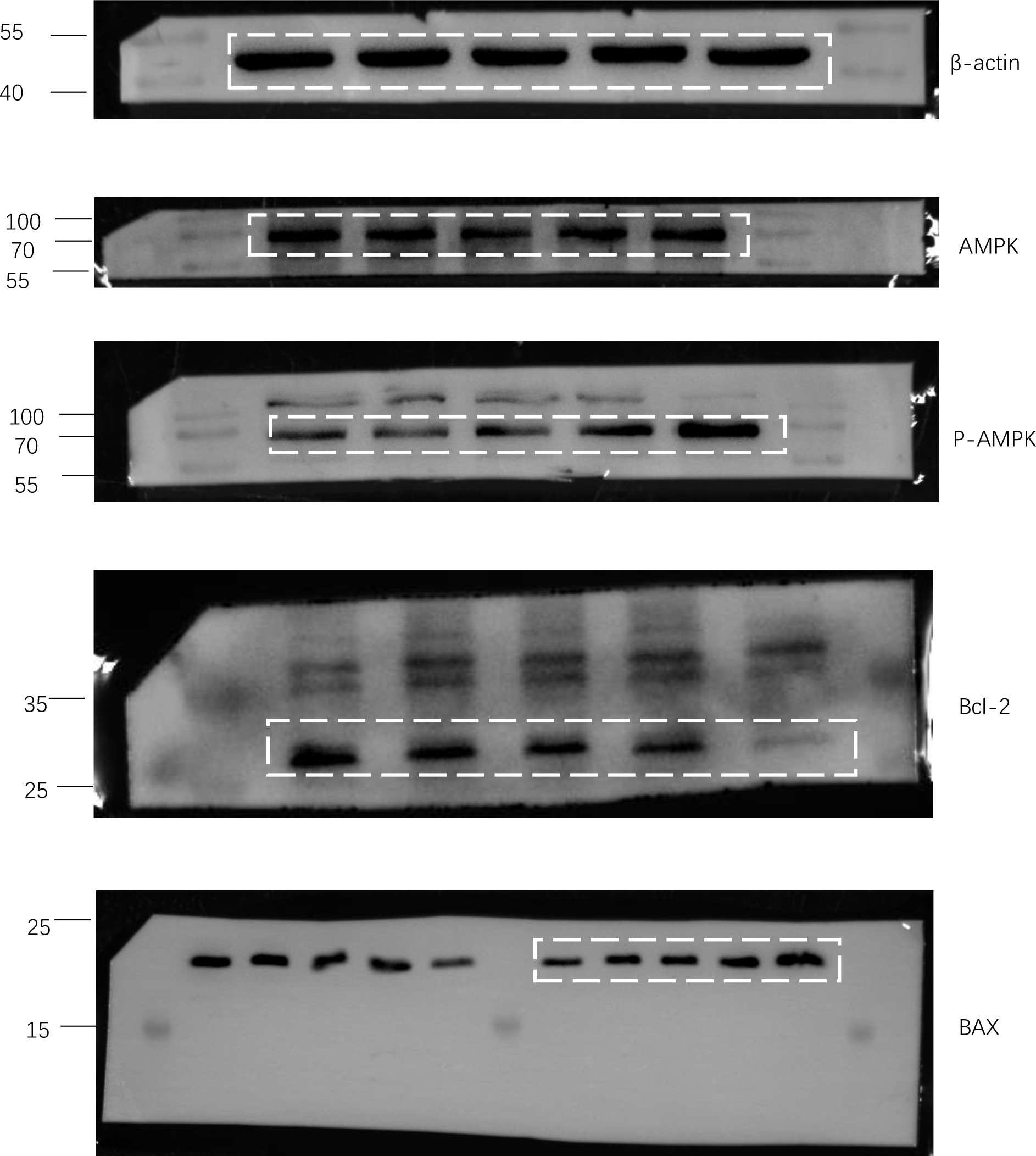
**

**(b)**

Fig S11. The original images of western blot analysis in Figure 4g. The white rectangles indicated the position of bands cropped from the original images.


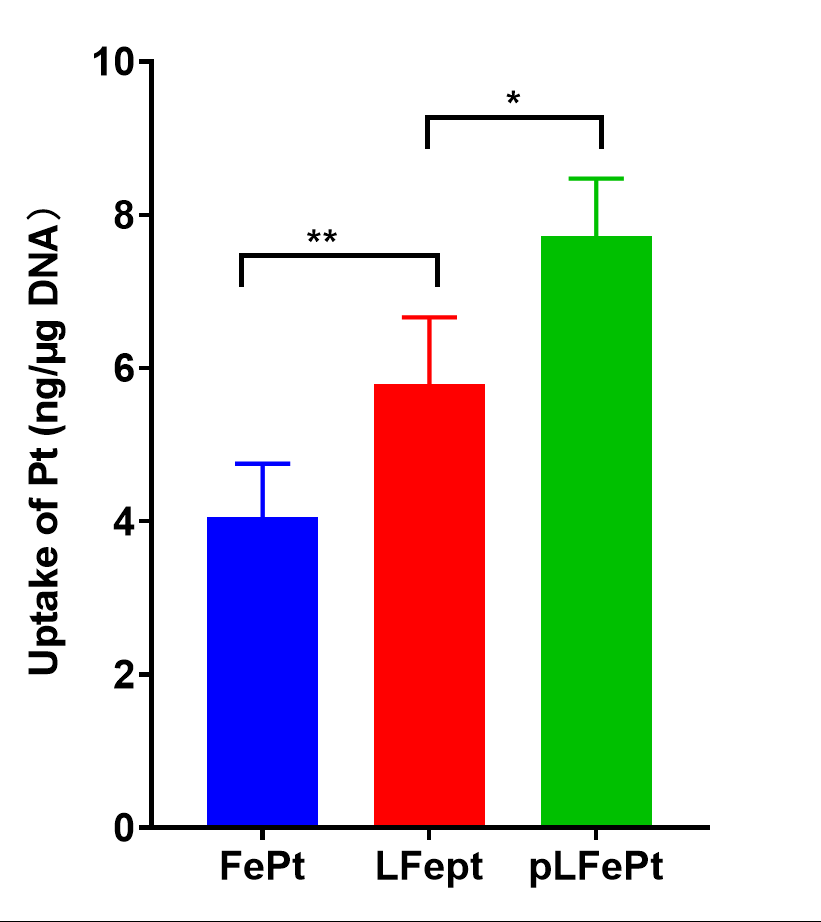


Fig S12. Pt-DNA adduct content of 4T1 cells after incubation with FePt, and pLFePt at an equivalent concentration for 6 h.


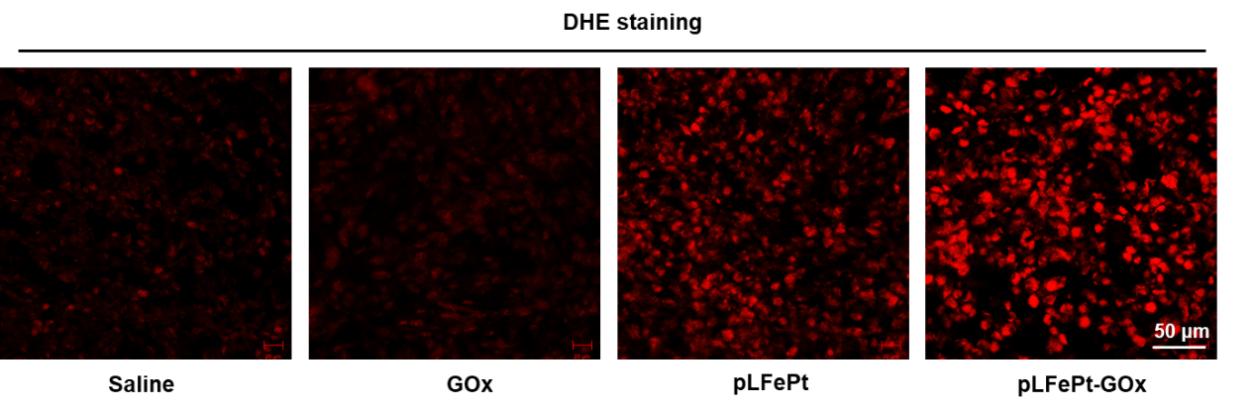


Fig S13. In vivo ROS-staining images of the tumors in different treatment groups.
